# TFAP2C promotes stemness and chemotherapeutic resistance in colorectal cancer via inactivating hippo signaling pathway

**DOI:** 10.1186/s13046-018-0683-9

**Published:** 2018-02-13

**Authors:** Xu Wang, Di Sun, Jiandong Tai, Si Chen, Miao Yu, Dong Ren, Lei Wang

**Affiliations:** 1 Department of Colorectal and Anal Surgery, The First Hospital of Jilin University, 71 Xinmin Street, Changchun, Jilin 130000 People’s Republic of China; 2 Center for Private Medical Service and Healthcare, The First Hospital of Sun Yat-sen University, Guangzhou, Guangdong 510080 China; 3 Department of Orthopaedic Surgery, The First Affiliated Hospital of Sun Yat-sen University, Guangzhou, 510080 China

**Keywords:** TFAP2C, Chemotherapeutic resistance, Cancer stem cells, Hippo signaling and colorectal cancer

## Abstract

**Background:**

Aberrant expression of transcription Factor AP-2 Gamma (TFAP2C) has been reported to be implicated in malignant process of many cancers. The purpose of this study is to investigate the clinical significance and biological roles of TFAP2C in colorectal cancer (CRC).

**Methods:**

TFAP2C expression was evaluated by real-time PCR, Western blot and immunohistochemistry (IHC) respectively in clinical CRC tissues. Statistical analysis was performed to explore the correlation between TFAP2C expression and clinicopathological features, and overall and progression-free survival in CRC patients. In vitro and in vivo *assays* were performed to assess the biological roles of TFAP2C in CRC cells. Western blot, luciferase and Chromatin immunoprecipitation (ChIP) assays were used to identify the underlying pathway mediating the biological roles of TFAP2C in CRC.

**Results:**

TFAP2C is robustly upregulated in CRC tissues and cells, and high expression of TFAP2C correlates with advanced clinicopathological features, poor prognosis and disease progression in CRC patients. Furthermore, upregulating TFAP2C enhances spheroids formation ability, the fraction of SP cells, expression of stem cell factors and the mitochondrial potential, and reduces the apoptosis induced by 5-fluorouracil in colorectal cancer cells in vitro, and promotes stemness and chemoresistance of CRC cells in vivo; while silencing TFAP2C yields an opposite effect. Importantly, downregulation of TFAP2C dramatically restores chemotherapeutic sensitivity of CRC cells to 5-FU in vivo. Our results further demonstrate that TFAP2C promotes stemness and chemoresistance of CRC cells to 5-FU by inhibiting Hippo signaling via transcriptionally upregulating ROCK1 and ROCK2 in CRC cells.

**Conclusion:**

Our findings indicate that TFAP2C may serve as a novel prognostic factor in CRC patients, and a therapeutic target for the treatment of CRC, suggesting that silencing TFAP2C in combination with 5-FU may be an effective therapeutic strategy to improve survival in CRC patients.

**Electronic supplementary material:**

The online version of this article (10.1186/s13046-018-0683-9) contains supplementary material, which is available to authorized users.

## Background

Colorectal cancer (CRC) is the most common malignant cancer in digestive tract worldwide [1, 2]. Despite great progress in treatment of CRC, including 5-fluorouracil (5FU)-based chemotherapy, the combination strategies of 5-FU and oxaliplatin or irinotecan, the five-year survival rate is still dismal [3, 4]. The existence of cancer stem cells (CSCs) that are the minority population of cells characterized by the capabilities of self-renewal, unlimited proliferation and differentiation into the multiple lineages of cancer cells has been regarded to be responsible for the failure of chemotherapy in CRC patients [5, 6], which contributes to the poor prognosis of CRC patients [7]. Indeed, multiple studies have shown that CSCs play crucial roles in the induction and maintenance of chemotherapeutic resistance in several human cancers [8, 9]. Thus, improved understanding of the mechanisms that maintain CSCs properties will improve the efficacy of chemotherapy in patients with CRC via eradicating the CSC population.

Activator protein-2 (AP-2) factors are a conserved family of DNA-binding transcription factors in different species and five members have been identified in mammals, including AP-2α-ɛ [10]. AP-2 factors play important roles in embryogenesis, and interestingly AP-2 factors expression is hardly detectable in most adult tissues [11, 12]. However, overexpression of AP-2 factors has been observed in multiple human cancers. For example, TFAP2A expression was elevated in nasopharyngeal carcinoma, which promoted nasopharyngeal carcinoma proliferation and growth via inducing cyclooxygenase-2 expression [13]; in addition, TFAP2B was reported to be overexpressed in non-small cell lung cancer (NSCLC) tissues and cell lines. TFAP2B knockdown by siRNA significantly attenuated cell growth and induced apoptosis in NSCLC cells in vitro and in a lung cancer subcutaneous xenograft model; conversely, upregulation of TFAP2B yielded an opposite effect [14]. Furthermore, accumulating literatures have shown that AP-2 proteins play crucial roles in the development of therapeutic resistance in the treatment of cancers. TFAP2A was elevated in breast cancer tissue and cell lines and more highly expressed in tamoxifen resistant tumor tissues and cell lines [15]. High expression of TFAP2C in breast cancer contributed to hormone resistance, which positively correlated with poor survival in breast cancer patients [16]. Moreover, low expression of TFAP2E due to hypermethylation was significantly associated with nonresponse to chemotherapy in colorectal cancer [17]. Therefore, these studies have indicated that different members of AP-2 proteins have stimulatory or inhibitory affect on chemotherapeutic response in cancer treatment, which may be environment and tumor type dependent.

The Hippo signaling pathway has been reported to be dysregulated in several biological processes and tumorigenesis of multiple human cancers [18–20]. In the mammal, the Hippo pathway constitutes of four core kinase cassette components, including kinases MST1/2 and LATS1/2, as well as the adaptor proteins SAV1 and MOB1 [20]. The Hippo signaling is active via tightly balancing the activity of YAP and TAZ, to low levels through phosphorylation– ubiquitination mechanisms [21, 22]. While Hippo signaling is absent, unphosphorylated YAP1/TAZ enters the nucleus and induce the transcriptional activity of TEA domain (TEAD) family members (TEAD1–TEAD4) as the transcriptional co-activators, which further transcriptionally upregulate multiple downstream effectors to exert a pleiotropic role in tumor progression and metastasis [23–25]. Moreover, accumulating studies has shown that the inactivation of Hippo signaling rendered resistance of cancer cells to chemotherapeutic drugs in various types of cancers. Chen et al. reported that simultaneous downregulation of MST1, LATS2, MOB1 and SAV1 by upregulation of miR-183c contributed to chemoresistance in pancreatic cancer [26]; in addition, the study by Touil and colleagues showed that hyper-activation of YAP promoted resistace of colon cancer cells to 5-FU [27]. Accordingly, the inactivation of Hippo pathway is considered as a crucial mediator in development of cancer chemoresistance, and better understanding of the mechanisms underlying the inactivation of Hippo pathway may provide new insights for the development of more effective cancer therapy.

In this study, we find that TFAP2C is significantly upregulated in CRC tissues and cells and high expression of TFAP2C correlates with advanced clinicopathological features, poor prognosis and disease progression in CRC patients. Furthermore, upregulation of TFAP2C enhances, while silencing TFAP2C inhibits CSCs characteristics and chemotherapeutic resistance in CRC cells in vitro *and* in vivo. Our results further reveal that TFAP2C promotes CSCs characteristics and chemoresistance via transcriptionally activating negative regulators of Hippo signaling, ROCK1 and ROCK2, resulting in inactivation of Hippo signaling in CRC cells. Therefore, our findings identify TFAP2C as a prognostic factor for CRC patients, as well as a therapeutic target to attenuate chemoresistance of CRC.

## Methods

### Cell lines and cell culture

The normal colon epithelial cell CMEC was purchased from PriCells, and all colorectal cancer cell lines, including RKO, CW-2, SW948, HCT116, SW480 COLO 210DM and COLO 205, were obtained from Shanghai Chinese Academy of Sciences cell bank (China). All were cultured in RPMI-1640 medium (Life Technologies, Carlsbad, CA, US) supplemented with penicillin G (100 U/ml), streptomycin (100 mg/ml) and 10%fetal bovine serum (FBS, Life Technologies) and cultured at 37 °C in a humidified atmosphere with 5% CO_2_.

### Patients and tumor tissues

A total of eight paired fresh colorectal cancer tissues with matched adjacent normal tissues and individual 378 paraffin-embedded, archived CRC tissues were obtained during surgery at the The First Hospital of Jilin University (Changchun, China) between January 2008 and December 2011 (Additional file 1: Table S1 and Additional file 2: Table S2). Patients were diagnosed based on clinical and pathological evidence, and the specimens were immediately snap-frozen and stored in liquid nitrogen tanks. For the use of these clinical materials for research purposes, prior patients’ consents and approval from the Institutional Research Ethics Committee were obtained.

### RNA extraction, reverse transcription, and real-time PCR

Total RNA from tissues or cells was extracted using TRIzol (Life Technologies) according to the manufacturer’s instructions. Messenger RNA (mRNA) were polyadenylated using a poly-A polymerase-based First-Strand Synthesis kit (TaKaRa, DaLian, China) and reverse transcription (RT) of total mRNA was performed using a PrimeScript RT Reagent kit (TaKaRa) according to the manufacturer’s protocol. Complementary DNA (cDNA) was amplified and quantified on ABI 7500HT system (Applied Biosystems, Foster City, CA, USA) using SYBR Green I (Applied Biosystems). Additional file 3: Table S3 lists the primers used in the reactions. Real-time PCR was performed according to a standard method, as described previously [28]. Primers for TFAP2C were synthesized and purified by RiboBio (Guangzhou, China). Glyceraldehyde-3-phosphate dehydrogenase (GAPDH) was used as endogenous controls for miRNA or mRNA respective. Relative fold expressions were calculated with the comparative threshold cycle (2^-ΔΔCt^) method according to the previous study [29].

### Plasmid, small interfering RNA and transfection

Human TFAP2C cDNA was purchased form (Vigene Biosciences, Shandong, China) and cloned into the pSin-EF2 plasmid (addgene #16578, Cambridge, MA, USA). Knockdown of endogenous TFAP2C was performed by cloning two short hairpin RNA (shRNA) oligonucleotides into the pSUPER-puro-retro vector (OligoEngine, Seattle, WA, USA). The full sequence and two separate shRNA fragments of TFAP2C are listed in Additional file 4: Table S4. The luciferase reporter system of pGL6-TA promoter-luc was used to examine the transcriptional activity of ROCK1 and ROCK2, and the sequences of ROCK1 and ROCK2 promoter was presented in Additional file 4: Table S4. Small interfering RNA (siRNA) for YAP and TAZ knockdown was obtained from Ribobio (Guangzhou, China). Transfection of siRNAs and plasmids was performed using Lipofectamine 3000 (Life Technologies) according to the manufacturer’s instructions.

### Western blotting analysis

Nuclear/cytoplasmic fractionation was separated by using Cell Fractionation Kit (Cell Signaling Technology, USA) according to the manufacturer’s instructions, and the whole cell lysates were extracted using RIPA Buffer (Cell Signaling Technology). Western blot was performed according to a standard method, as described previously [30]. Antibodies against TFAP2C, BCL2 and BCL2L1, p-MST1/2, p-LATS1, LATS1, p-YAP, YAP, ROCK1 and ROCK2 were purchased from Cell Signaling Technology (Cambridge, USA), and MST1 and TAZ from Abcam. The membranes were stripped and reprobed with an anti–α-tubulin antibody (Cell Signaling Technology) as the loading control.

### Chromatin immunoprecipitation (ChIP)

Cells were cultured as described above. Cross-linking was performed with formaldehyde (Merck) at a final concentration of 1% and terminated after 5 min by the addition of glycine at a final concentration of 0.125 M. Cells were harvested with SDS buffer, pelleted by centrifugation, and resuspended in IP buffer. Chromatin was sheered by sonication (HTU SONI 130; Heinemann) to generate DNA fragments with an average size of 500 bp. Preclearing and incubation with anti-Flag (F1804, Sigma) antibodies or IgG control (M-7023, Sigma) for 16 h was performed as previously described (Menssen et al. 2007). Washing steps and the reversal of cross-linking were performed as described previously (Frank et al. 2001). Immunoprecipitated DNA was analyzed by qPCR and the primers of ROCK1 and ROCK2 promoter was presented in Additional file 3: Table S3. Enrichment was expressed as the percentage of the input for each condition.

### Anchorage-independent growth assay

A total of 500 cells were trypsinized and suspended in 2 ml of complete medium containing 0.3% agar (Sigma). This experiment was performed as previously described [31] and carried out three times independently for each cell line.

### Cell counting Kit-8 analysis

For cell counting kit-8 analysis, cells (2 × 10^3^) were seeded into 96 well plates and the specific staining process and methods were performed according to the previous study [32].

### Colony formation assay

The cells were trypsinized as single cell and suspended in the media with 10% FBS. The indicated cells (300 cells per well) were seeded into of 6-well plate for ~ 10–14 days. Colonies were stained with 1% crystal violet for 10 min after fixation with 10% formaldehyde for 5 min. Plating efficiency was calculated as previously described [33]. Different colony morphologies were captured under a light microscope (Olympus).

### Flow cytometric analysis

Flow cytometric analyzed of apoptosis were using the FITC Annexin V Apoptosis Detection Kit I (BD, USA), and was performed previously described [34]. The cell’s inner mitochondrial membrane potential (Δψm) was detected by flow cytometric using MitoScreen JC-1 staining kit (BD), and was presented as protocol described. Briefly, cells were dissociated with trypsin and resuspended at 1 × 10^6^ cells/ml in Assay Buffer, and then incubated at 37 °C for 15 min with 10 μl/ml JC-1. Before analyzed by flow cytometer, cells were washed twice by Assay Buffer. Flow cytometry data were analyzed using FlowJo 7.6 software (TreeStar Inc., USA).

### Caspase-9 or Caspase-3 activity assays

Activity of caspase-9 or caspase-3 was analysis by spectrophotometry using Caspase-9 Colorimetric Assay Kit or Caspase-3 Colorimetric Assay Kit (Keygen, China), and was presented as protocol described. Briefly, 5 × 10^6^ cells or 100 mg fresh tumor tissues were washed with cold PBS and resuspended in Lysis Buffer and incubated on ice for 30 min. Mixed the 50 μl cell suspension, 50 μl Reaction Buffer, and 5 μl Caspase-3/− 9 substrate, and then incubated at 37 °C for 4 h. The absorbance was measured at 405 nm, and BCA protein quantitative analysis was used as the reference to normal each experiment groups.

### Side population analysis

The cell suspensions were labeled with Hoechst 33,342 (Molecular probes – #H-.

3570) dye for side population analysis as per standard protocol [35]. Briefly, cells were resuspended at 1× pre-warmed OptiMEM (Gibco, USA) containing 2% FBS (Gibco, USA) at a density of 10^6^/mL. Hoechst 33,342 dye was added at a final concentration of 5 lg/mL in the presence or absence of verapamil (50 lmol/L; Sigma) and the cells were incubated at 37 °C for 90 min with intermittent shaking. At the end of the incubation, the cells were washed with OptiMem containing 2% FBS and centrifuged down at 4 °C, and resuspended in ice-cold OptiMem containing 2% FBS and 10 mmol/L HEPES. Propidium iodide (Sigma, USA) at a final concentration of 2 lg/mL was added to the cells to gate viable cells. The cells were filtered through a 40-lm cell strainer to obtain single cell suspension before sorting. Analysis and sorting was done on a FACS AriaI (Becton Dickinson). The Hoechst 33,342 dye was excited at 355 nm and its dual-wavelength emission at blue and red region was plotted to get the SP scatter.

### Spheroid formation assay

Cells (500 cells/well) were seeded into 6-well Ultra Low Cluster plates (Corning) and cultured as previously described [36]. After 10–12 days, the number of cell spheroids (tight, spherical, non-adherent masses > 50 μm in diameter) were counted, and images of the spheroids were scored under an inverse microscope (spheroids formation efficiency = colonies/input cells× 100%).

### Tumor xenografts

Four-week-old BALB/c-nu female mice weighing 15–20 g were maintained in a standard pathogen-free environment where the animals were housed in sterile cages under laminar flow hoods in a 20–26 °C temperature controlled room with a 12-h light/12-h dark cycle and fed autoclaved chow and water. The 6-week-old BALB/c-nu mice were randomly divided into four groups (*n* = 6 per group). Cells (1 × 10^6^, 1 × 10^5^ 1 × 10^4^ and 1 × 10^3^) were inoculated subcutaneously together with Matrigel (final concentration of 25%) into the inguinal folds of the nude mice respectively. To study the effect of TFAP2C on chemoresistance of CRC cells, The 4–6 week-old BALB/c-nu mice were randomly divided into four groups (n = 6 per group) and the indicated cells (2 × 10^6^) were inoculated subcutaneously into the inguinal folds of the nude mice. After seven days for cells inoculation, the mice were injected intraperitoneally 50 mg/kg.d 5-FU for 4 weeks. Tumor volume was determined using an external caliper and calculated using the eq. (L × W2)/2. On day 38, animals were euthanized, tumors were excised, weighed and stored in liquid nitrogen tanks.

### Immunohistochemistry

The immunohistochemistry procedure and scoring of TFAP2C expression levels were performed as previously described [37]. Scores given by two independent investigators were averaged for further comparative evaluation of TFAP2C expression. The proportion of tumor cells was scored as follows: 0 (no positive tumor cells); 1 (< 10% positive tumor cells); 2 (10–35% positive tumor cells); 3 (35–70% positive tumor cells) and 4 (> 70% positive tumor cells). The staining intensity was graded according to the following criteria: 0 (no staining); 1 (weak staining, light yellow); 2 (moderate staining, yellow brown) and 3 (strong staining, brown). The staining index (SI) was calculated as the product of the staining intensity score and the proportion of positive tumor cells. Using this method of assessment, we evaluated TFAP2C expression in CRC samples by determining SI, with scores of 0, 1, 2, 3, 4, 6, 8, 9 or 12. An SI score 4 was the median SI of all sample tissues. High and low TFAP2C expression was stratified by the follow criteria: SI ≤ 4 was used to define tumors with low TFAP2C expression; SI ≥6 was used to define tumors with high TFAP2C expression.

### Luciferase assay

Cells (4 × 10^4^) were seeded in triplicate in 24-well plates and cultured for 24 h, and the luciferase reporter assay was performed as previously described [38]. Cells were transfected with 100 ng pROCK1 or ROCK2 promoter reporter luciferase plasmid, plus 5 ng pRL-TK Renilla plasmid (Promega) using Lipofectamine 3000 (Invitrogen) according to the manufacturer’s recommendation. Luciferase and Renilla signals were measured 36 h after transfection using a Dual Luciferase Reporter Assay Kit (Promega) according to the manufacturer’s protocol.

### Immunofluorescence

Cells were seeded on glass culture slides (BD Biosciences) and fixed with 4% cold methanol at − 20 °C for 10 min. Subsequently, cells were blocked with 10% goat serum for 1 h and incubated with primary antibodies against YAP (Cell Signaling Technology, Cat. 14,074), TAZ (Abcam, Cat. ab224239) or Phalloidin-iFluor 488 Reagent - CytoPainter (Abcam, Cat. ab176753) at room temperature for 2 h and then incubated with secondary antibody FITC for 1 h at room temperature. After counterstained with DAPI (Invitrogen), the slide was observed under a confocal microscope (Zeiss).

### Statistical analysis

All values are presented as means ± standard deviation (SD). Significant differences were determined using GraphPad 5.0 software (USA). Student’s t-test was used to determine statistical differences between two groups. One-way ANOVA was used to determine statistical differences between multiple testing. The chi-square test was used to analyze the relationship between TFAP2C expression and clinicopathological characteristics. Survival curves were plotted using the Kaplan Meier method and compared by log-rank test. *P* < 0.05 was considered significant. All the experiments were repeated three times.

## Results

### TFAP2C is upregulated in colorectal cancer cell lines and tissues

**Fig. 1:**
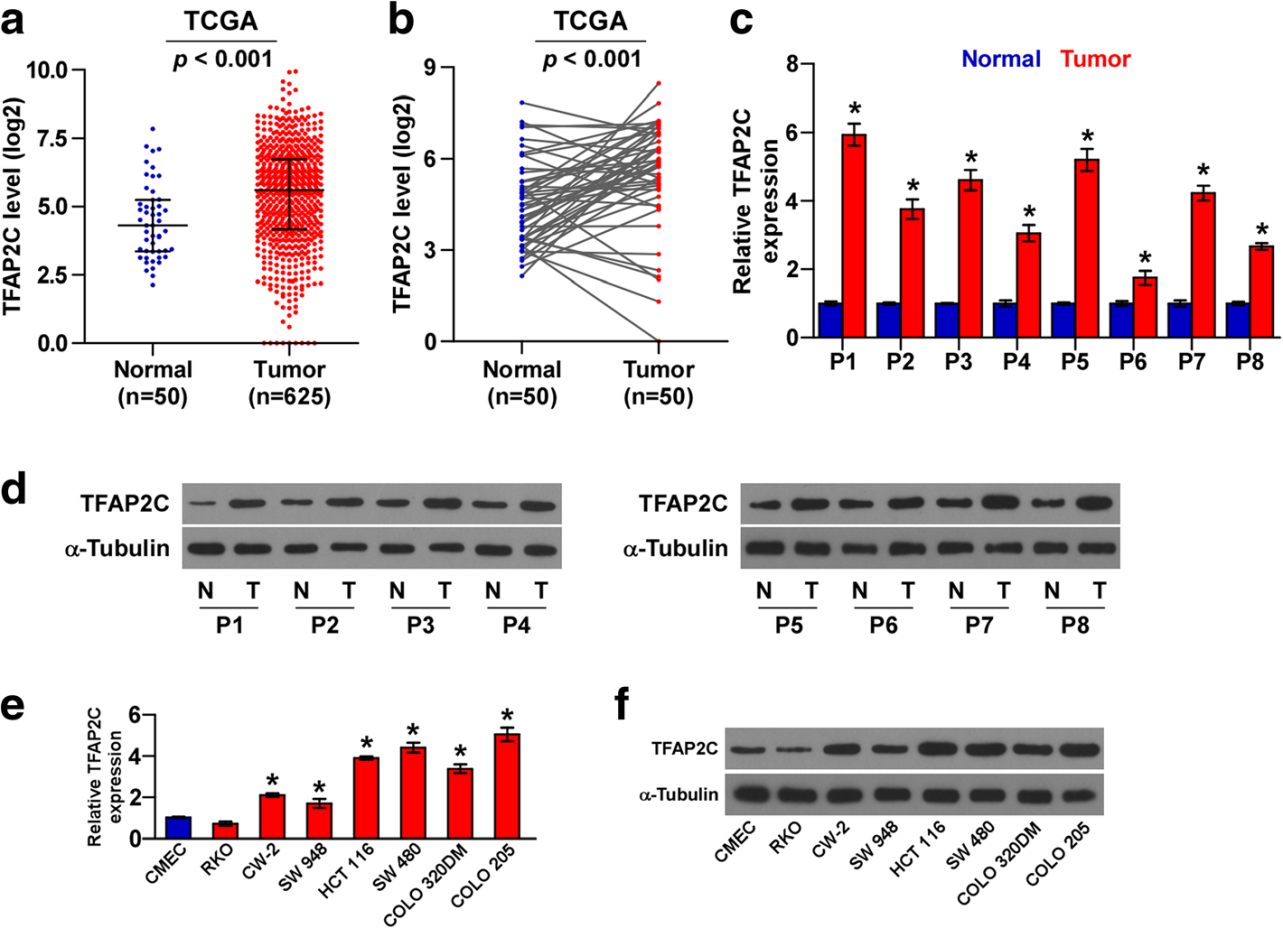
TFAP2C is upregulated in CRC tissues and cells. **a** TFAP2C expression level was elevated in colorectal cancer tissues compared with the adjacent normal tissues as assessed by analyzing the TCGA colorectal cancer RNA sequencing dataset (Adjacent normal tissue, *n* = 50; Colorectal cancer, *n* = 625). **b** TFAP2C expression level was elevated in 50 paired colorectal cancer tissues compared with the adjacent normal tissues as assessed by analyzing the TCGA colorectal cancer RNA sequencing dataset. **c** and **d** Real-time PCR analysis and Western blotting of TFAP2C expression in 8 paired CRC tissues. α-Tubulin was detected as a loading control in the Western blot and GAPDH was used as endogenous controls in RT-PCR. Each bar represents the mean values ± SD of three independent experiments. *P < 0.05. **e** and **f** Real-time PCR and Western blotting analysis of TFAP2C expression in one normal colon epithelial cell CMEC and 7 CRC cell lines. GAPDH was used as endogenous controls in RT-PCR and α-Tubulin was detected as a loading control in the Western blot. Each bar represents the mean values ± SD of three independent experiments. *P < 0.05

Through analyzing several colorectal cancer RNA sequencing datasets from The Cancer Genome Atlas (TCGA) and ArrayExpress, we found that TFAP2C expression was dramatically elevated in CRC tissues compared with the adjacent normal colorectal tissues (Fig. 1a and Additional file 5: Figure S1). The TFAP2C expression level in 50 paired primary CRC tissue samples from TCGA was further analyzed and the result showed that TFAP2C expression in the majority of CRC tissues was higher than their matched adjacent normal tissue samples (Fig. 1b). We further validated TFAP2C expression in our own 8 paired colorectal cancer tissues and found that mRNA and protein expression elevels of TFAP2C was differentially upregulated in CRC tissue samples compared with the matched adjacent normal tissue samples (Fig. 1c and d). Consistently, expression levels of TFAP2C were upregulated in CRC cells compared with normal colon epithelial cell CMEC, except RKO cell (Fig. 1e and f). Thus, these results indicate that overexpression of TFAP2C contributes to the progression of colorectal cancer.

### Overexpression of TFAP2C correlates with advanced clinico0pathological features and poor prognosis in CRC patients

**Fig. 2:**
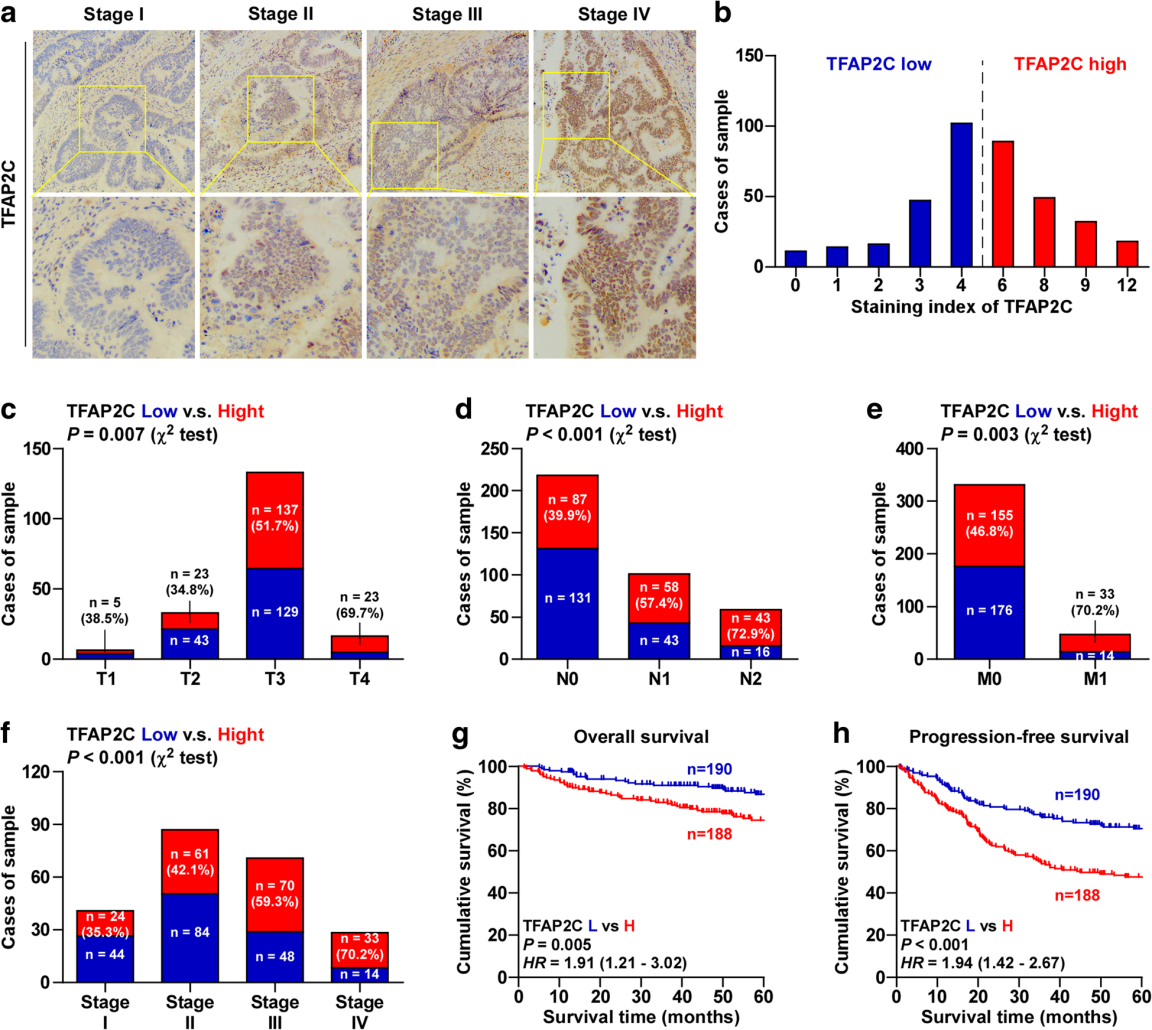
Overexpression of TFAP2C correlates with advanced clinicopathological features and poor prognosis in CRC patients. **a** Representative images of TFAP2C expression in CRC tissues of different clinical stages. **b** The number of CRC tissues stratified by staining index of IHC. **c** Percentages and number of samples showed high or low TFAP2C expression in CRC tissues with different tumor volume. **d** Percentages and number of samples showed high or low TFAP2C expression in CRC tissues with different lymph node metastasis status. **e** Percentages and number of samples showed high or low TFAP2C expression in CRC tissues with different distant metastasis status. **f** Percentages and number of samples showed high or low TFAP2C expression in CRC tissues with different stage. **g** and **h** Kaplan-Meier overall survival and progression-free survival curves for CRC patients stratified by high and low expression of TFAP2C

We further examined TFAP2C expression by immunohistochemical analysis of 378 human CRC tissues (Additional file 2: Table S2) and found TFAP2C expression was primarily detected within the cytoplasm and nucleus, and the expression levels of TFAP2C positively correlated with clinical stages (Fig. 2a). High expression of TFAP2C was observed in 188/378 CRC tissues (49.7%) (Fig. 2b). In addition, there were potent positive associations between TFAP2C expression and T classification, N classification, M classification and clinical stage (Fig. 2c-f and Additional file 6: Table S5). Kaplan-Meier survival analysis indicated that CRC patients with high TFAP2C expression exhibited shorter overall survivals and progression-free survivals (Fig. 2g and h), which were further verified by the analysis results from several publicly available CRC dataset (Additional file 7: Figure S2A-F). Collectively, these results demonstrate that the overexpression of TFAP2C correlates with advanced clinicopathological characteristics, and poor prognosis and disease progression in CRC patients.

### Overexpression of TFAP2C confers chemotherapeutic resistance in CRC cells

Interestingly, we found that high expression of TFAP2C positively with poor chemotherapy response in CRC patients via analyzing CRC datasets from TCGA and GSE28702 (Additional file 8: Figure S3A and B), which was further validated in our CRC tissues (Additional file 8: Figure S3C). We further analyzed the correlation of TFAP2C expression with chemotherapeutic response in CRC cells via calculating the apoptotic ratio of CRC cells under treatment of 5-FU, and found that CRC cells with high expression level of TFAP2C displayed more resistance to 5-FU (Additional file 8: Figure S3D). Furthermore, negative correlation of TFAP2C expression with apoptotic ration was found in CRC cells (Additional file 8: Figure S3E and F). These finding suggest that high expression level of TFAP2C may be associated with chemoresistance in CRC.

**Fig. 3:**
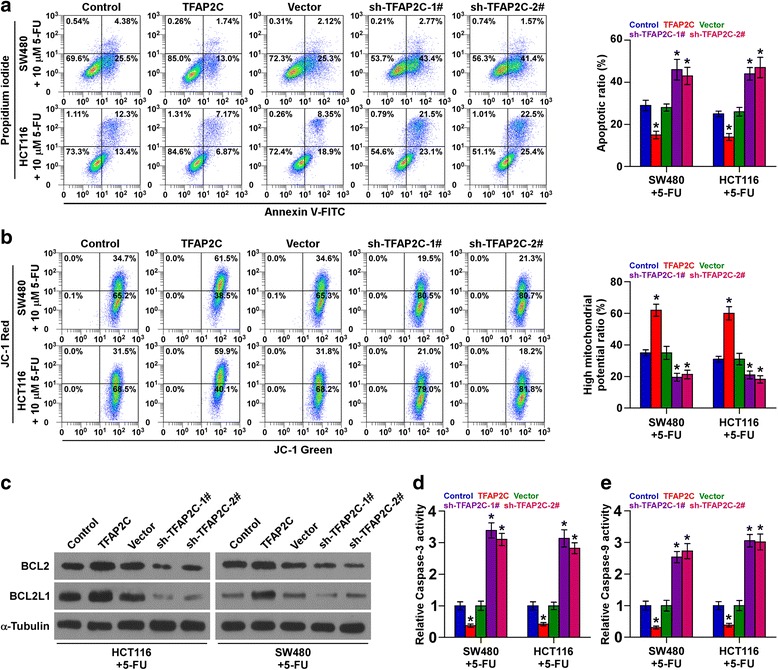
Upregulation TFAP2C promotes chemoresistance in CRC cells in vitro. **a** Annexin V-FITC/PI staining of the indicated cells treated with 5-FU (10 μM) for 36 h. Error bars represent the mean ± S.D. of three independent experiments. *P < 0.05. **b** The JC-1 staining showed that upregulating TFAP2C increased, while silencing TFAP2C reduced the mitochondrial potential in the indicated CRC cells. Error bars represent the mean ± S.D. of three independent experiments. *P < 0.05. **c** Western blotting analysis of BCL2 and BCL2L1 in the indicated cells. α-Tubulin was detected as a loading control. **d** and **e** Analysis of the activities of caspase-3 (**d**) and caspase-9 (**e**) were detected by the cleaved forms of these two proteins. Error bars represent the mean ± S.D. of three independent experiments. *P < 0.05

To determine the stimulatory roles of TFAP2C in the chemotherapeutic resistance of CRC, we first constructed TFAP2C-stably expressing SW480 and HCT116 CRC cell lines by ectopically overexpressing TFAP2C and endogenously knocking down TFAP2C via retrovirus infection (Additional file 9: Figure S4A and B). As shown in Fig. 3a, upregulating TFAP2C reduced, while silencing TFAP2C increased the apoptosis rate of CRC cells treated with 5-FU. Moreover, TFAP2C overexpression enhanced, while silencing TFAP2C decreased, the mitochondrial potential of CRC cells under treatment of 5-FU (Fig. 3b). We further examined the effect of TFAP2C on the expression levels of the anti-apoptotic proteins BCL2 and BCL2L1 and found upregulating TFAP2C increased, while silencing TFAP2C reduced BCL2 and BCL2L1 expression (Fig. 3c). The effect of TFAP2C on the activity of caspase-3 or − 9 was further investigated and the results revealed that upregulating TFAP2C repressed the activity of caspase-3 or − 9; conversely, silencing TFAP2C increased the activity of caspase-3 or − 9 (Fig. 3d and e). Furthermore, our results demonstrated that silencing TFAP2C dramatically reduced the cell proliferation ability via CCK-8, colony formation and anchorage-independent growth assays; however, upregulating TFAP2C had no obvious effect on the proliferation ability of CRC cells (Additional file 9: Figure S4C-E), suggesting that overexpression of TFAP2C promotes chemoresistance of CRC cells to 5-FU independent on the effects of TFAP2C on cell proliferation. Collectively, these results indicate that TFAP2C promotes chemoresistance of CRC cells to 5-FU in vitro.

### Silencing TFAP2C restores chemosensitivity of CRC cells to 5-FU in vivo

**Fig. 4:**
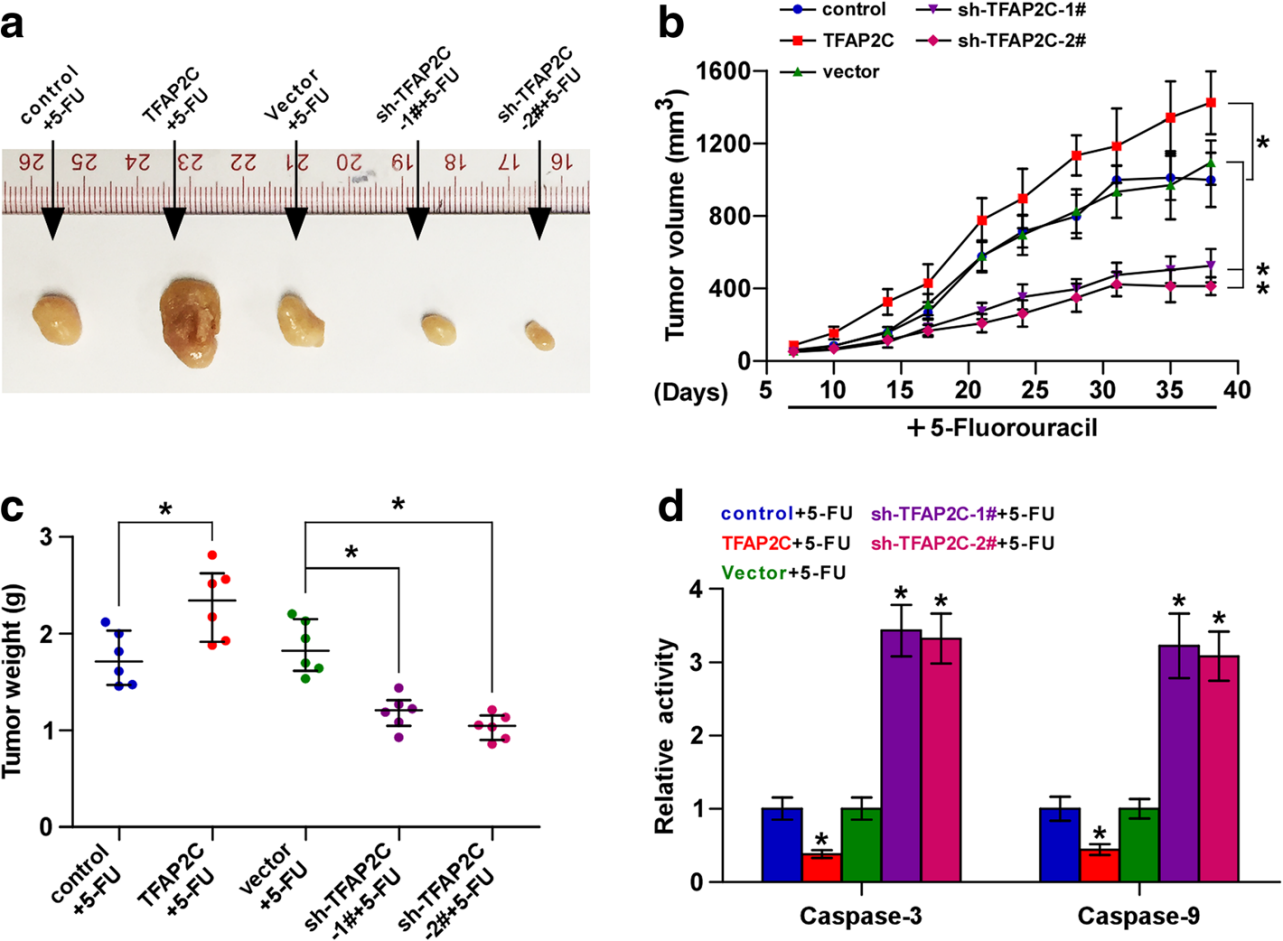
Downregulation of TFAP2C improves chemosensitivity of CRC cells to 5-FU in vivo. **a** Representative images of the tumors are shown in the xenograft model of nude mice. **b** After one weeks of inoculating HCT116 cells, mice were intraperitoneal injected with 50 mg/kg 5-FU per day for four weeks. Tumor volumes in the indicated groups were measured from the fifth day at five days interval. Data presented are the mean ± s.d. **c** Tumor weights of each group. **d** Analysis of the activities of caspase-3 and caspase-9 were examined in the indicated tumor from mice. Error bars represent the mean ± S.D. of three independent experiments. *P < 0.05

We further examined the effect of TFAP2C on the chemoresistance of CRC cells in vivo. Mice were randomly divided into five groups (*n* = 6/group) and 2 × 10^6^ HCT116 cells of control, TFAP2C-overexpression, vector, TFAP2C-RNAi#1 and TFAP2C-RNAi#2 were inoculated subcutaneously in the left dorsal flank respectively. A weeks later, each group of mice were injected intraperitoneally with 5-FU (50 mg/kg.d). As shown in Fig. 4a-c, the tumor volumes and weight were increased in the TFAP2C -overexpressing plus 5-FU mice group, but were dramatically decreased in the TFAP2C-silencing plus 5-FU mice group, compared to the corresponding mice controls. Furthermore, the activity of caspase-3 or − 9 in the tumor tissues from TFAP2C -overexpressing plus 5-FU mice group was robustly suppressed, but enhanced in the tumor tissues from TFAP2C -silencing plus 5-FU mice group (Fig. 4d). Collectively, these findings indicate that silencing TFAP2C improves chemotherapeutic sensitivity of CRC cells to 5-FU in vivo.

### TFAP2C promotes cancer stem characteristics in CRC cells

**Fig. 5:**
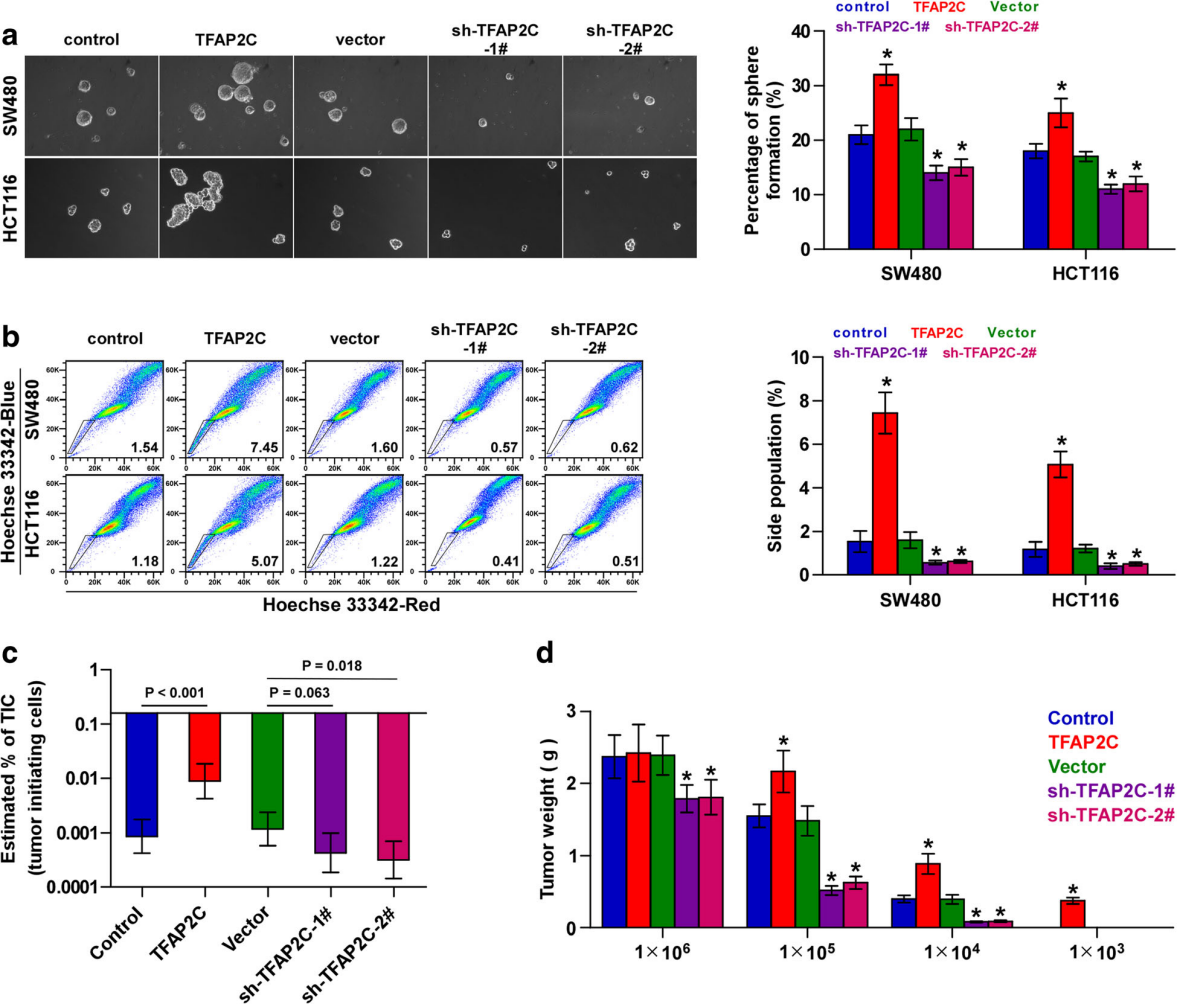
TFAP2C promotes cancer stem cell characteristics in CRC cells. **a** Representative images of spheroids formed at 200-fold magnification were counted. Histograms showed the mean number of spheroids formed. Error bars represent the mean ± S.D. of three independent experiments. *P < 0.05. Scale bar: 100 μm. **b** Hoechst 33,342 dye exclusion assay showed that upregulating TFAP2C promoted, while silening TFAP2C decreased the fraction of side population. Error bars represent the mean ± S.D. of three independent experiments. *P < 0.05. **c** The estimated percentage of tumor initiating cells required in the indicated mice group. **d** Tumor weights of the indicate mice group

Several lines of evidence have shown that cancer stem cells (CSCs) are crucial for the induction and maintenance of chemotherapeutic resistance in cancers [8, 9], which is responsible for the failure of chemotherapy in cancer treatment, suggesting that these two cellular processes are intimately linked. Therefore, we further evaluated the effects of TFAP2C on CSCs characteristics in CRC cells. Spheroids formation assay was performed and the results showed that overexpression of TFAP2C increased spheroids formation ability of CRC cells, while silencing TFAP2C decreased spheroids formation ability (Fig. 5a). Side population (SP) analysis further revealed that upregulating TFAP2C enhanced, while silencing TFAP2C decreased the fraction of SP cells (Fig. 5b). The effects of TFAP2C on expression levels of stem cell factors, including NANOG, BMI-1, OCT4 and SOX2, were further examined, and the results showed that upregulating TFAP2C enhanced, while silencing TFAP2C reduced the expression of these stem cell factors (Additional file 10: Figure S5A and B).

The effect of TFAP2C on tumorigenesis of CRC cells was further investigated in vivo. As shown in Fig. 5c, the number of tumor initiating cells (TIC) required to develop tumor in mice were significantly reduced in the TFAP2C-overexpressing mice group, but enhanced in TFAP2C-silenced mice group. Furthermore, the tumors formed by TFAP2C-overexpressing cells were larger than tumors in the control group after implantation of 1 × 10^6^, 1 × 10^5^ or 1 × 10^4^ cells; conversely, tumors weight in the TFAP2C-silencing group were lower than the tumors in the control group (Fig. 5d). Importantly, tumors were only detected after inoculation of 1 × 10^3^ TFAP2C-overexpressing cells (Fig. 5d and Additional file 10: Figure S5C). Collectively, these findings indicate that TFAP2C promotes CSCs characteristics of CRC cells in vitro and in vivo.

### TFAP2C inactivates hippo signaling pathway in CRC cells

**Fig. 6:**
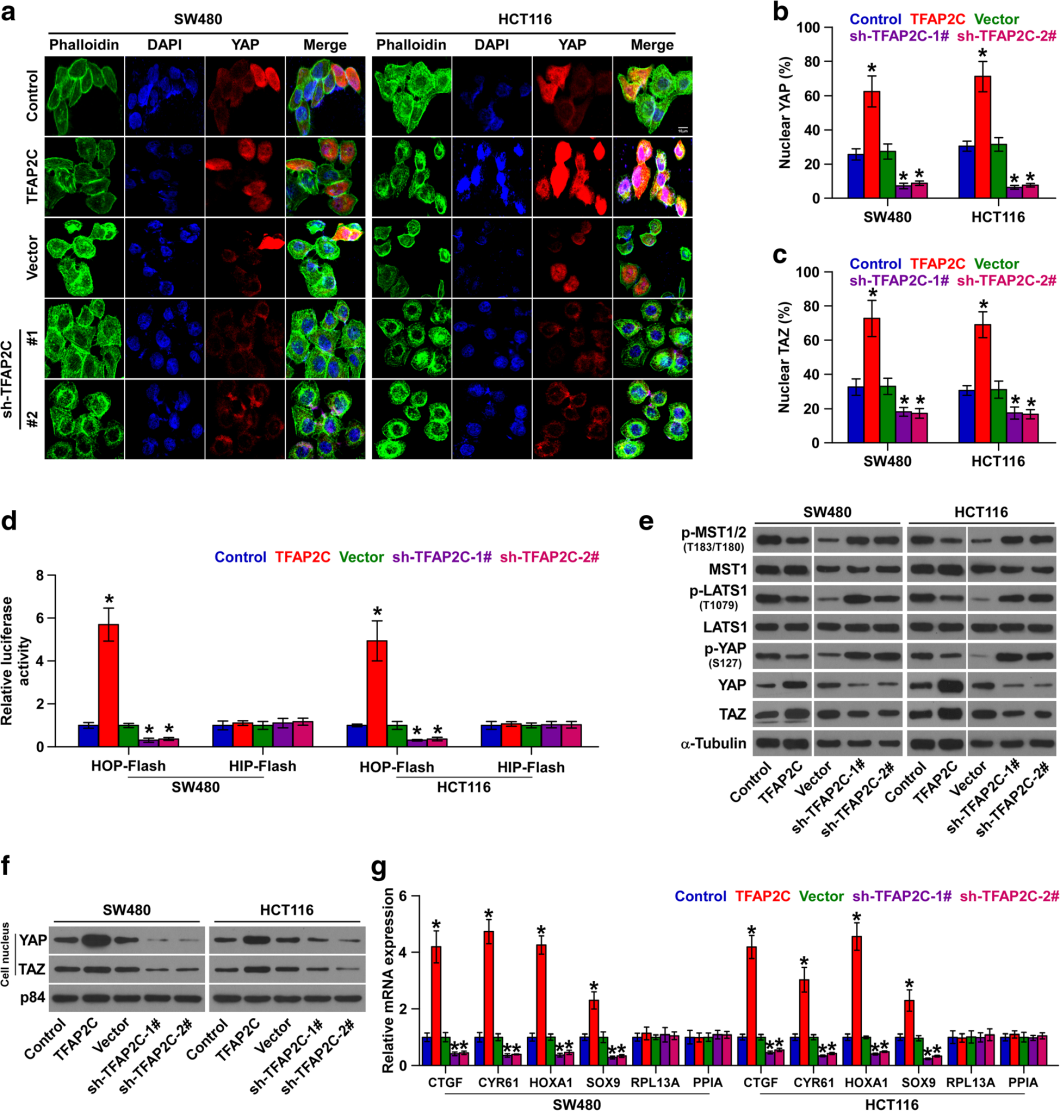
TFAP2C inactivates Hippo signaling pathway in CRC cells. **a** Representative immunofluorescent images of CRC cells were immunostained with YAP antibody (red) or phalloidin (green) in the indicated CRC cells. **b** and **c** The percentage of nuclear YAP+ (**b**) and nuclear TAZ+ (**c**) cell number via immunostaining in the indicated groups. *P < 0.05. **d** TEAD transcriptional activity was assessed by HOP-Flash luciferase reporter in the indicated cells. Error bars represent the mean ± S.D. of three independent experiments. *P < 0.05. **e** Western blotting of p-MST1/2, MST1/2, p-LATS1, LAST1, p-YAP, YAP and TAZ expression in the indicated cells. α-tubulin was used as the loading control. **f** Cellular fractionation and western blotting analysis of nuclear YAP and TAZ expression in the indicated cells. The nuclear protein p84 was used as the nuclear protein marker. **g** Real-time PCR analysis of CTGF, CYR61, HOXA1, SOX9, RPL13A and PPIA in the indicated cells. Error bars represent the mean ± S.D. of three independent experiments. *P < 0.05

To determine the underlying signaling pathways mediating the stimulatory effects of TFAP2C on chemoresistance and stemness in CRC cells, luciferase reporter plasmids of multiple signaling pathways were transfected into CRC cells respectively, and we found that transcriptional activity of TEAD, the transcriptional co-activators of Hippo signaling pathway, was the most highly regulated by upregulation of TFAP2C, but silenced by downregulation of TFAP2C in CRC cells (Additional file 11: Figure S6A). GSEA was further performed and the results showed that TFAP2C expression level was positively associated with the YAP and TAZ-activated gene signatures, suggesting that the inactivation of Hippo signaling pathway may be associated with the pro-tumor effects of TFAP2C (Additional file 11: Figure S6B and C). Analysis of CRC datasets from TCGA revealed that expression levels of TFAP2C positively correlated with the protein expression levels of transcriptional co-activators YAP and TAZ of Hippo signaling (Additional file 11: Figure S6D-G). Immunofluorescence staining demonstrated that upregulating TFAP2C enhanced, while silencing TFAP2C reduced the nuclear translocation of YAP and TAZ (Fig. 6a-c). Luciferase assay showed that upregulating TFAP2C significantly enhanced, while silencing TFAP2C repressed, TEAD-dependent luciferase activity in CRC cells (Fig. 6d). Moreover, cellular fractionation and western blotting analysis revealed that overexpression of TFAP2C reduced the p-MST1/2, p-LATS1 and p-YAP levels, increased cytoplasmic and nuclear YAP and TAZ levels, and had no effect on total expression level of MST1/2 and LATS1 in CRC cells; however, silencing TFAP2C yielded an opposite effects (Fig. 6e and f). Real-time PCR analysis showed that upregulation of TFAP2C increased, while silencing TFAP2C decreased expression levels of multiple downstream genes, including CTGF, CYR61, HOXA1 and SOX9 in CRC cells, but had no effect on expression levels of house-keeping genes RPL13A and PPIA (Fig. 6g). Thus, these results demonstrate that TFAP2C inactivates Hippo signaling pathway in CRC cells.

### TFAP2C promotes chemoresistance and stemness via inactivating hippo signaling

We further determine whether activity of Hippo signaling is involved in the effects of TFAP2C on chemoresistance and stemness in CRC cells, and found that individual silencing of YAP or TAZ attenuated the stimulatory effects of TFAP2C overexpression on sphere formation, SP fraction and mitochondrial potential in CRC cells (Additional file 12: Figure S7A–C). Furthermore, apoptotic ratio of CRC cells repressed by TFAP2C overexpression was dramatically reversed by silencing YAP or TAZ (Additional file 12: Figure S7D). Taken together, these findings indicate that TFAP2C promotes the chemoresistance and stemness via inactivating Hippo signaling in CRC cells.

### TFAP2C promotes stemness and chemoresistance of CRC cells via transcriptionally activating ROCK1 and ROCK2

**Fig. 7:**
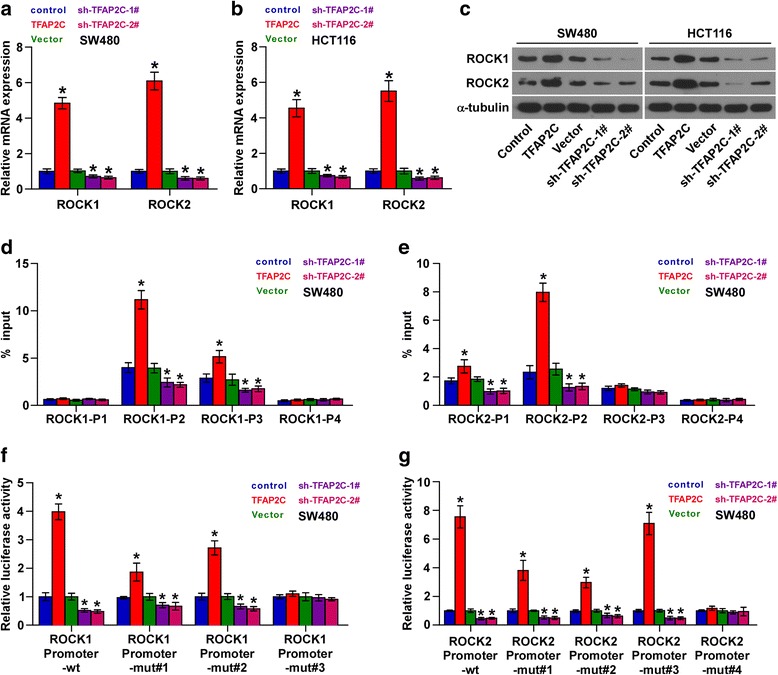
TFAP2C transcriptionally upregulates ROCK1 and ROCK2 in CRC cells. **a**-**c** Real-time PCR analysis and Western blotting of ROCK1 and ROCK2 expression in the indicated CRC cells. α-Tubulin was detected as a loading control in the Western blot and GAPDH was used as endogenous controls in RT-PCR. Each bar represents the mean values ± SD of three independent experiments. *P < 0.05. **d** and **e** Analysis of ROCK1 and ROCK2 promoters physically associated with TFAP2C by using chromatin immunoprecipitation (ChIP) assay in the indicated SW480 cells. *P < 0.05. **f** and **g** Relative luciferase activity of the indicated promoter vectors in SW480 cells transfected with TFAP2C plasmids or siRNA. *P < 0.05

Through analyzing JASPAR, we found several TFAP2C-binding motifs inside the putative promoter region of ROCK1 and ROCK2 (Additional file 13: Figure S8A and B), both of which have been reported to be negative regulators of Hippo signaling pathway via inhibition of LATS activity [39]. The UCSC bioinformatics identified four potential binding sites of TFAP2C in the promoter region of ROCK1 and ROCK2 (Additional file 13: Figure S8C and D). RT-PCR and Western blot analysis showed that upregulation of TFAP2C enhanced, while silencing TFAP2C reduced mRNA and protein expression levels of ROCK1 and ROCK2 (Fig. 7a-c). A ChIP assay indicated that TFAP2C could bind to the P2 and P3 binding sites in the promoter region of ROCK1 and P1 and P2 binding sites in ROCK2 in CRC cells (Fig. 7d and e, and Additional file 14: Figure S7A and B). Furthermore, an enhancement of the ROCK1 and ROCK2 promoter luciferase activity was observed on upregulation of TFAP2C in CRC cells; conversely, downregulation of TFAP2C significantly reduced the luciferase activity (Fig. 7f and g, and Additional file 14: Figure S7C and D). However, upregulation or downregulation of TFAP2C had no effect on the luciferase activity of the ROCK1 and ROCK2 promoter that contained all mutations in the P2 and P3 binding sites (Fig. 7a, b, g and h, and Additional file 12: Figure S7E and F). Importantly, Y-27632, a specific inhibitor of ROCK1 and ROCK2, significantly repressed stemness and chemoresistance in TFAP2C-overexpressing cells (Additional file 15: Figure S10A-D), as well as increased levels of p-MST1/2, p-LATS1 and p-YAP and decreased total and nuclear levels of YAP and TAZ (Additional file 15: Figure S10E-I). Collectively, our results indicate that TFAP2C promotes stemness and chemotherapeutic resistance of CRC cells via transcriptionally activating ROCK1 and ROCK2, leading to inactivation of Hippo signaling pathway in CRC cells.

### Clinical relation of TFAP2C with hippo signaling activity in human CRC tissues

To determine the clinical correlation of TFAP2C with Hippo signaling activity in clinical CRC tissues, the protein levels of TFAP2C, p-MST1/2, p-LATS1, YAP and TAZ expression were examined in four 5-FU resistant and four 5-FU sensitive CRC tissues. As shown in Additional file 16: Figure S11A-F, TFAP2C, YAP and TAZ expression levels were upregulated in resistant CRC tissues (T1–4) compared with that in sensitive CRC tissues (T5–8); conversely, p-MST1/2 and p-LATS1 expression were reduced in resistant CRC tissues. Thus, our results indicate that there is a positive clinical correlation of TFAP2C with inactivation of Hippo signaling in CRC tissues.

## Discussion

In the current study, we found that TFAP2C was upregulated in CRC tissues and cells, which correlated with advanced clinicopathological features, poor prognosis and disease progression in CRC patients. Furthermore, upregulation of TFAP2C enhanced, while silencing TFAP2C attenuated stemness and chemotherapeutic resistance in CRC cells in vitro and in vivo. Our results further reveal that TFAP2C promoted CSCs characteristics and chemoresistance via transcriptionally upregulating ROKC1 and ROCK2 expression, leading to inactivation of Hippo signaling. Therefore, our findings uncover a novel mechanism by which TFAP2C promotes CSCs characteristics and chemoresistance in CRC.

Accumulating studies have shown that aberrant expression of AP-2 proteins was implicated in the development, progression and metastasis in several human cancers. For example, TFAP2C expression was elevated in lung carcinoma and high expression of TFAP2C promoted cell cycle activation and lung carcinoma cell tumorigenesis via by upregulating the oncogenic miR-183 and downregulating tumor-suppressive miRNA-33a [40]. Moreover, low TFAP2B expression was reported in primary neuroblastomas, which correlated with poor prognosis via promoting proliferation and cell cycle progression [41]. Furthermore, different members of AP-2 proteins play opposite roles in the same tumor type. In breast cancer, TFAP2A functioned as a tumor suppressor; conversely, TFAP2C played an oncogenic role in the development and progression of breast cancer [42]. These studies indicated that although belonged to the AP-2 family, different members of AP-2 proteins function as either oncogene or tumor suppressor in cancers. In the current study, we found that TFAP2C was remarkably elevated in CRC tissues and high expression of TFAP2C correlated with advanced clinicopathological features, poor prognosis and disease progression in CRC patients. Furthermore, upregulation of TFAP2C enhanced the chemotherapeutic resistance in CRC cells; conversely silencing TFAP2C yielded an opposite effect. Therefore, our findings indicate that TFAP2C plays an oncogenic role in CRC via promoting chemoresistance and stemness of CRC cells.

TFAP2C has been reported to be upregulated in various types of cancer, including lung carcinoma, breast cancer, and high expression of TFAP2C significantly correlated with poor prognosis via promoting the growth and proliferation [40, 43, 44]. However, other studies have shown that TFAP2C played a tumor suppressive role in several human cancers, such as melanoma, endometrial cancer [45, 46]. Interestingly, Bogachek and colleagues reported that TFAP2C regulated multiple breast cancer-related genes, and loss of TFAP2C induced epithelial-mesenchymal transition in breast cancer cells [47]. These studies suggest that the pro- and anti-tumor roles of TFAP2C are function and tumor type dependent. However, the clinical significance and biological role of TFAP2C in colorectal cancer remain largely unknown. In this study, high expression of TFAP2C was observed in CRC tissues, which correlated with advanced clinicopathological features, poor prognosis and disease progression in CRC patients. Moreover, our results demonstrated that TFAP2C promoted the chemoresistance and stemness of CRC cells in vitro and in vivo, further determining the tumor-stimulatory role of TFAP2C in CRC.

Numerous studies have reported that several regulatory mechanisms were responsible for the inactivation of Hippo signaling, which played an important role in the chemotherapeutic resistance of cancer. Studies have consistently shown that inactivation of Hippo signaling by downregulation of the Hippo pathway components mammalian MST1/2 andLATS1/2 contributed to resistance of cancer cells to chemotherapeutic drugs [48–50]. Furthermore, upregulation of YAP or TAZ conferred chemotherapeutic resistance in multiple cancer types [51–53]. Recently, it was reported that Rho-associated protein kinase (ROCK) repressed activity of Hippo signaling through inhibition of LATS activity and cell polarity in the outside cells [39]. Importantly, Cao and colleagues reported that TFAP2C inhibited Hippo signaling dependent on ROCK activity [54]. It remains unclear, however, how ROCK mediates the inhibitory effect of TFAP2C on Hippo signaling activity and whether TFAP2C/ROCK/Hippo signaling promotes chemotherapeutic resistance in CRC. In this study, our results revealed that TFAP2C transcriptionally activated ROCK1 and ROCK2 via binding to the promoter region of ROCK1 and ROCK2 in CRC cells. Importantly, the stimulatory effects of TFAP2C on chemoresistance and stemness in CRC cells were effectively attenuated by the specific inhibitor of ROCK1 and ROCK2, Y-27632. Thus, our results uncover a novel mechanism by which TFAP2C promotes chemotherapeutic resistance and stemness in CRC cells.

It has been widely documented that AP-2 members may be used as a prognostic marker in numerous human tumor types. A study by Ikram et al. showed that low TFAP2B expression caused by CpG methylation of the TFAP2B locus in primary neuroblastomas significantly promoted proliferation and cell cycle progression and low expression of TFAP2B significantly associated with unfavorable prognostic markers as well as adverse patient outcome [41]. Conversely, TFAP2A and TFAP2B correlated with good overall and disease-free survival in breast cancer patients [55]. These studies indicated that different AP-2 proteins predict different prognosis in different tumor types. Furthermore, TFAP2C has been extensively reported to be a poor prognostic marker in several human cancers, including breast cancer, lung carcinoma, [16, 40, 44]. However, the correlation of TFAP2C with prognosis of CRC patients remains unknown. In this study, our results found that TFAP2C expression level was increased in CRC tissues and high expression of TFAP2C correlated with advanced clinicopathological features. More importantly, Kaplan-Meier survival analysis revealed that CRC patients with high TFAP2C expression exhibited shorter overall survivals and progression-free survivals. Thus, these findings identify TFAP2C as a potential prognostic marker in CRC patients.

## Conclusion

In summary, our findings demonstrate that TFAP2C inactivates Hippo signaling via transcriptionally upregulating ROCK1 and ROCK2 expression, which further promotes chemotherapeutic resistance and stemness in CRC. Therefore, better understanding the underlying mechanism and the specific role of TFAP2C in the pathogenesis of CRC facilitates the development of novel therapeutic strategies for treatment of CRC.

## Additional files

Additional file 1: **Table S1.** The basic information of 8 colorectal cancer patients for TFAP2C mRNA and protein expression analysis. (PDF 51 kb)

Additional file 2: **Table S2.** The basic information of 378 patients with colorectal cancer for TFAP2C immunohistochemical staining analysis. (PDF 55 kb)

Additional file 3: **Table S3.** A list of primers used in the reactions for real-time RT-PCR. (PDF 61 kb)

Additional file 4: **Table S4.** A list of primers used in the reactions for clone PCR. (PDF 49 kb)

Additional file 5: **Table S5.** The relationship between TFAP2C IHC expression level and clinical. (PDF 61 kb)

Additional file 6: **Figure S1.** TFAP2C expression level was elevated in colorectal cancer tissues compared with the benign colorectal lesions as assessed by analyzing the GSE17538 colorectal cancer RNA sequencing dataset (Benign, *n* = 6; Colorectal cancer, *n* = 232).(PDF 35 kb)

Additional file 7: **Figure S2.** Overexpression of TFAP2C is associated poor overall and progression-free survivals in CRC patients (A-C) Overall survival curves from the TCGA, GSE17538 and GSE38832 profiles for CRC patients stratified by high and low expression of TFAP2C. (D-F) Progression-free survival curves from the TCGA, GSE17538 and GSE38832 profiles for CRC patients stratified by high and low expression of TFAP2C. (PDF 233 kb)

Additional file 8: **Figure S3.** Overexpression of TFAP2C is associated with poor chemotherapy response. (A and B) TFAP2C expression levels were much higher in CRC patients with poor chemotherapy response as assessed by analyzing the TCGA and GSE28702 CRC RNA sequencing datasets. (C) Percentages and number of samples showed high or low TFAP2C expression in CRC patients with different chemotherapy response in our CRC tissues. (D) Apoptotic ratio of CRC cells under treatment of 5-FU (20μm). (E and F) The correlation of TFAP2C mRNA (E) and protein (F) expression levels with apoptotic ratio in CRC cells after treated with 20μm 5-FU. (PDF 166 kb)

Additional file 9: **Figure S4.** Silencing TFAP2C inhibits proliferation ability of CRC cells. (A and B) Real-time PCR and Western blot of the indicated CRC cells transfected with TFAP2C -vector, TFAP2C, TFAP2C -RNAi-vector, TFAP2C -RNAi#1 and TFAP2C -RNAi#2. GAPDH was used as endogenous controls in RT-PCR and α-Tubulin was detected as a loading control in the Western blot. Each bar represents the mean values ± SD of three independent experiments. *P < 0.05. (C) CCK-8 assay revealed that silencing TFAP2C decreased the proliferation rate in CRC cells. Each bar represents the mean values ± SD of three independent experiments. *P < 0.05. (D) downregulation of endogenous TFAP2C reduced, the mean colony number in the colony formation assay. Each bar represents the mean values ± SD of three independent experiments. *P < 0.05. (E) Representative micrographs and colony numbers in the indicated group in the anchorage-independent growth assay. Each bar represents the mean values ± SD of three independent experiments. *P < 0.05. (PDF 167 kb)

Additional file 10: **Figure S5.** (A and B) Real-time PCR analysis of OCT4A, SOX2, NANOG and BMI-1 expression in the indicated cells. GAPDH was used as the loading control. Error bars represent the mean ± S.D. of three independent experiments. *P < 0.05. (C) The formation number of tumor initiated by different amounts of HCT116 cells in nude mice. (PDF 106 kb)

Additional file 11: **Figure S6.** (A) Activity of luciferase reporter constructs of several signaling pathway were examined in the TFAP2C-overexpressing or –silencing CRC cells. (B and C) TFAP2C expression level was positively associated with the YAP and TAZ-activated gene signatures. (D-G) TFAP2C expression level is positively associated with the protein expression levels of transcriptional co-activators YAP and TAZ of Hippo signaling pathway as assessed through CRC dataset from TCGA. (PDF 162 kb)

Additional file 12: **Figure S7.** (A and B) Individual silencing of YAP or TAZ attenuated the sphere formation ability and SP fraction in the TFAP2C-overexpressing CRC cells. *P < 0.05. (C and D) Individual silencing of YAP or TAZ reversed the effects of TFAP2C upregulation on mitochondrial potential and apoptotic ratio in CRC cells. *P < 0.05. (PDF 99 kb)

Additional file 13: **Figure S8.** (A-B) The putative binding sites of TFAP2C in ROCK1 and ROCK2 promoters by JASPAR. (C and D) Schematic representation of the promoter regions of ROCK1 and ROCK2 with the putative TFAP2C binding sites through UCSC. (PDF 171 kb)

Additional file 14: **Figure S9.** (A and B) Analysis of ROCK1 and ROCK2 promoters physically associated with TFAP2C by using chromatin immunoprecipitation (ChIP) assay in the indicated HCT116 cells. *P < 0.05. (C and D) Relative luciferase activity of the indicated promoter vectors in the indicated HCT116 cells. *P < 0.05. (PDF 135 kb)

Additional file 15: **Figure S10.** (A-D) The specific inhibitor of ROCK1 and ROCK2, Y-27632, significantly repressed SP fraction, sphere formation ability, mitochondrial potential and BCL2, BCL2L1 expression in the TFAP2C-overexpressing CRC cells. (E and F) Representative immunofluorescent images of CRC cells were immunostained with YAP or TAZ antibody (red) or phalloidin (green) in the indicated CRC cells. (G and H) The percentage of nuclear TAZ+ (G) and nuclear YAP+ (H) cell number via immunostaining in the indicated groups. *P < 0.05. (I) Western blotting of ROCK1, ROCK2, p-MST1/2, MST1/2, p-LATS1, LAST1, p-YAP, YAP and TAZ expression, and nuclear YAP and TAZ expression in the indicated cells. α-tubulin and p84 were used as the loading control for cytoplasm and nucleus respectively. (PDF 383 kb)

Additional file 16: **Figure S11.** Clinical relation of TFAP2C with Hippo signaling activity in human CRC tissues. (A) Analysis of TFAP2C expression with p-MST1/2, p-LATS1, YAP and TAZ expression in 4 resistant CRC tissues (T1-4) and 4 sensitive CRC tissues (T5-8). α-tubulin was used as loading control. (B-F) Relative expression levels of TFAP2C, p-MST1/2, p-LATS1, YAP and TAZ expression in CRC tissues.The expression levels of TFAP2C, p-MST1/2, p-LATS1, YAP and TAZ expression were quantified by densitometry using Image J, and normalized to the levels of α-tubulin respectively. The sample with the lowest expression of each protein was used as a standard. (PDF 191 kb)

## Abbreviations

BMI1: BMI1 Proto-Oncogene, Polycomb Ring Finger
CTGF: Connective Tissue Growth Factor
CYR61: Cysteine Rich Angiogenic Inducer 61
HOXA1: Homeobox A1
IHC: Immunohistochemistry
NANOG: Nanog Homeobox
OCT4: POU Class 5 Homeobox 1
PCR: Polymerase Chain Reaction
ROCK1/2: Rho Associated Coiled-Coil Containing Protein Kinase 1/2
SOX2: SRY-Box 2
SOX9: SRY-Box 9
TAZ: WW Domain Containing Transcription Regulator 1
TCGA: The Cancer Genome Atlas
TEAD: TEA Domain Transcription Factor
TFAP2C: Transcription Factor AP-2 Gamma
YAP: Yes Associated Protein 1
