# Supplementary material for: TFAP2C promotes stemness and chemotherapeutic resistance in colorectal cancer via inactivating hippo signaling pathway
Source: J Exp Clin Cancer Res. 2018 Feb 13;37:27. doi: 10.1186/s13046-018-0683-9 (PMC5812206; doi:10.1186/s13046-018-0683-9)
Supplement: Supplementary file 1 — Table S1. The basic information of 8 colorectal cancer patients for TFAP2C mRNA and protein expression analysis. (PDF 51 kb) [file 13046_2018_683_MOESM1_ESM.pdf]

Table S1. The basic information of 8 colorectal cancer patients for TFAP2C mRNA and protein expression analysis.

		Cases (n)	Percentage (%)
Location	Colon	4	50.0
	Rectum	4	50.0
Gender	Male	4	50.0
	Female	4	50.0
Age	≤60	3	37.5
	>60	5	62.5
Grade	G1	0	0.0
	G2	8	100.0
	G3	0	0.0
Stage	Stage I	2	25.0
	Stage II	2	25.0
	Stage III	4	50.0
	Stage IV	0	0.0
