# Supplementary material for: TFAP2C promotes stemness and chemotherapeutic resistance in colorectal cancer via inactivating hippo signaling pathway
Source: J Exp Clin Cancer Res. 2018 Feb 13;37:27. doi: 10.1186/s13046-018-0683-9 (PMC5812206; doi:10.1186/s13046-018-0683-9)
Supplement: Supplementary file 2 — Table S2. The basic information of 378 patients with colorectal cancer for TFAP2C immunohistochemical staining analysis. (PDF 55 kb) [file 13046_2018_683_MOESM2_ESM.pdf]

Table S2. The basic information of 378 patients with colorectal cancer for TFAP2C immunohistochemical staining analysis.

		Cases (n)	Percentage (%)
Location	Colon	118	31.2
	Rectum	260	68.8
Gender	Male	169	44.7
	Female	209	55.3
Age	≤60	133	35.2
	>60	245	64.8
Grade	G1	34	9.0
	G2	307	81.2
	G3	37	9.8
T classification	T1	13	3.4
	T2	66	17.5
	T3	266	70.4
	T4	33	8.7
N classification	N0	218	57.7
	N1	101	26.7
	N2	59	15.6
M classification	M0	331	87.6
	M1	47	12.4
Stage	Stage I	68	18.0
	Stage II	145	38.4
	Stage III	118	31.2
	Stage IV	47	12.4
Chemotherapeutic	Sensitivity	39	23.6
response	Resistance	36	21.8
(Excluded Stage I/II)	Not available	90	54.5
