# Supplementary material for: TFAP2C promotes stemness and chemotherapeutic resistance in colorectal cancer via inactivating hippo signaling pathway
Source: J Exp Clin Cancer Res. 2018 Feb 13;37:27. doi: 10.1186/s13046-018-0683-9 (PMC5812206; doi:10.1186/s13046-018-0683-9)
Supplement: Supplementary file 3 — Table S3. A list of primers used in the reactions for real-time RT-PCR. (PDF 61 kb) [file 13046_2018_683_MOESM3_ESM.pdf]

Table 3. A list of primers used in the reactions for real-time RT-PCR.

Gene	Sequence (5`– 3`)
TFAP2C-up	TCAGTCCCTGGAAGATTGTCG
TFAP2C -dn	CCAGTAACGAGGCATTTAAGCA
GAPDH-up	TCCTCTGACTTCAACAGCGACAC
GAPDH-dn	CACCCTGTTGCTGTAGCCAAATTC
ROCK1-Point-1#-up	AAGAGAACAGCGCTACTCACC
ROCK1-Point-1#-dn	CACAGCAGCAGCAACATGTC
ROCK1-Point-2#-up	GGTCCCCGTCCCGAGATG
ROCK1-Point-2#-dn	TCACCCGCCCTTTGCTTTC
ROCK1-Point-3#-up	TGCTGATGGGGGAGCTTCTG
ROCK1-Point-3#-dn	CGGCGGTCTCCGTTTGTTTGA
ROCK1-Point-4#-up	GCAGCCAGTCCAGAGACTTC
ROCK1-Point-4#-dn	AAACGCCACTGGTGCTCAATG
ROCK2-Point-1#-up	GCGGCTCTTTGTCATGTACCC
ROCK2-Point-1#-dn	TGGCCATGCTTCATACACCAG
ROCK2-Point-2#-up	CTCCTCGCGCTCAGGTCCC
ROCK2-Point-2#-dn	GGGACGCCGTCTGTTCCC
ROCK2-Point-3#-up	GGACCCCGCGGACTACCC
ROCK2-Point-3#-dn	TGAGGCGAGGCCCGCATA
ROCK2-Point-4#-up	CAGCAGGGCTATTCACAATGTTC
ROCK2-Point-4#-dn	GTCTGCATACCTGCCAGGATTC
ROCK1-up	GGTGGTCGGTTGGGGTATTTT
ROCK1-dn	CGCCCTAACCTCACTTCCC
ROCK2-up	TCAGAGGTCTACAGATGAAGGC
ROCK2-dn	CCAGGGGCTATTGGCAAAGG
CTGF-up	TGGAGATTTTGGGAGTACGG

CTGF-dn	CAGGCTAGAGAAGCAGAGCC
CYR61-up	GGTCAAAGTTACCGGGCAGT
CYR61-dn	GGAGGCATCGAATCCCAGC
HOXA1-up	TCCTGGAATACCCCATACTTAGC
HOXA1-dn	GCACGACTGGAAAGTTGTAATCC
SOX9-up	AGCGAACGCACATCAAGAC
SOX9-dn	CTGTAGGCGATCTGTTGGGG
NANOG-up	TCCAACATCCTGAACCTCAGCTA
NANOG-dn	AGTCGGGTTCACCAGGCATC
SOX2-up	GTGAGCGCCCTGCAGTACAA
SOX2-dn	GCGAGTAGGACATGCTGTAGGTG
OCT4-up	TGAAGCTGGAGAAGGAGAAGCTG
OCT4-dn	GCAGATGGTCGTTTGGCTGA
BMI-1-up	TCGTTGTTCGATGCATTTCT
BMI-1-dn	CTTTCATTGTCTTTTCCGCC
RPL13A-up	GCCATCGTGGCTAAACAGGTA
RPL13A-dn	GTTGGTGTTCATCCGCTTGC
PPIA-up	GGCAAATGCTGGACCCAACACA
PPIA-dn	TGCTGGTCTTGCCATTCCTGGA
