# Supplementary material for: TFAP2C promotes stemness and chemotherapeutic resistance in colorectal cancer via inactivating hippo signaling pathway
Source: J Exp Clin Cancer Res. 2018 Feb 13;37:27. doi: 10.1186/s13046-018-0683-9 (PMC5812206; doi:10.1186/s13046-018-0683-9)
Supplement: Supplementary file 4 — Table S4. A list of primers used in the reactions for clone PCR. (PDF 49 kb) [file 13046_2018_683_MOESM4_ESM.pdf]

Table S4. A list of primers used in the reactions for clone PCR.

Gene	Sequence (5` – 3`)
TFAP2C-full-up	ATGTTGTGGAAAATAACCGATAATG
TFAP2C-full-dn	TTATTTCCTGTGTTTCTCCATTTTC
ROCK1-Pro-luci-up	CCCCGTCTTCCCCTCACTG
ROCK1-Pro-luci-dn	GCGATCAGAAGCAGCCAGAG
ROCK2-Pro-luci-up	AGCAAGCTCTCCACGTTGATG
ROCK2-Pro-luci-dn	GCTCGGTCGCTATTATTTGGGC
shTFAP2C-1#-up	GATCCCCGCCCAGCAACTGTGTAAAGAATTCAAGAG ATTCTTTACACAGTTGCTGGGCTTTTTA
shTFAP2C-1#-dn	AGCTTAAAAAGCCCAGCAACTGTGTAAAGAATCTCT TGAATTCTTTACACAGTTGCTGGGCGGG
shTFAP2C-2#-up	GATCCCCTGTTACCTACCTTACTATTTATTCAAGAGAT AAATAGTAAGGTAGGTAACATTTTTA
shTFAP2C-2#-dn	AGCTTAAAAATGTTACCTACCTTACTATTTATCTCTTG AATAAATAGTAAGGTAGGTAACAGGG
