# Supplementary material for: TFAP2C promotes stemness and chemotherapeutic resistance in colorectal cancer via inactivating hippo signaling pathway
Source: J Exp Clin Cancer Res. 2018 Feb 13;37:27. doi: 10.1186/s13046-018-0683-9 (PMC5812206; doi:10.1186/s13046-018-0683-9)
Supplement: Supplementary file 5 — Table S5. The relationship between TFAP2C IHC expression level and clinical. (PDF 61 kb) [file 13046_2018_683_MOESM5_ESM.pdf]

Table S5. The relationship between TFAP2C IHC expression level and clinical pathological characteristics in 378 patients with colorectal cancer.

Parameters	Number of cases	TFAP2C IHC expression		P values
		Low	High	
Location		190	188	
ADC	118	54	64	0.238
SQC	260	136	124	
Gender				
Male	169	79	90	0.216
Female	209	111	98	
Age				
<60	133	70	63	0.460
≥60	245	120	125	
Grade				
G1-G2	341	175	166	0.213
G3	37	15	22	
T classification				
T1-2	79	51	28	0.004*
T3-4	299	139	160	
N classification				
N0	218	131	87	<0.001*
N1-2	160	59	101	
M classification				
M0	331	176	155	0.003*
M1	47	14	33	
Stage				
I-II	213	128	85	<0.001*
III-IV	165	62	103	

Chemotherapeutic response				
Sensitivity	39	21	18	0.011*
Resistance	36	9	27	
