# Supplementary figures and images for: TFAP2C promotes stemness and chemotherapeutic resistance in colorectal cancer via inactivating hippo signaling pathway
Source: J Exp Clin Cancer Res. 2018 Feb 13;37:27. doi: 10.1186/s13046-018-0683-9 (PMC5812206; doi:10.1186/s13046-018-0683-9)

## Supplemental Figure 1

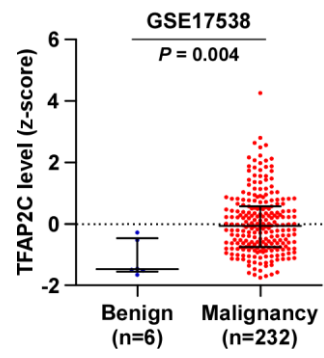

Supplement: Supplementary file 6 — Figure S1. TFAP2C expression level was elevated in colorectal cancer tissues compared with the benign colorectal lesions as assessed by analyzing the GSE17538 colorectal cancer RNA sequencing dataset (Benign, n = 6; Colorectal cancer, n = 232).(PDF 35 kb) [file 13046_2018_683_MOESM6_ESM.pdf]

**Supplemental Figure 2**

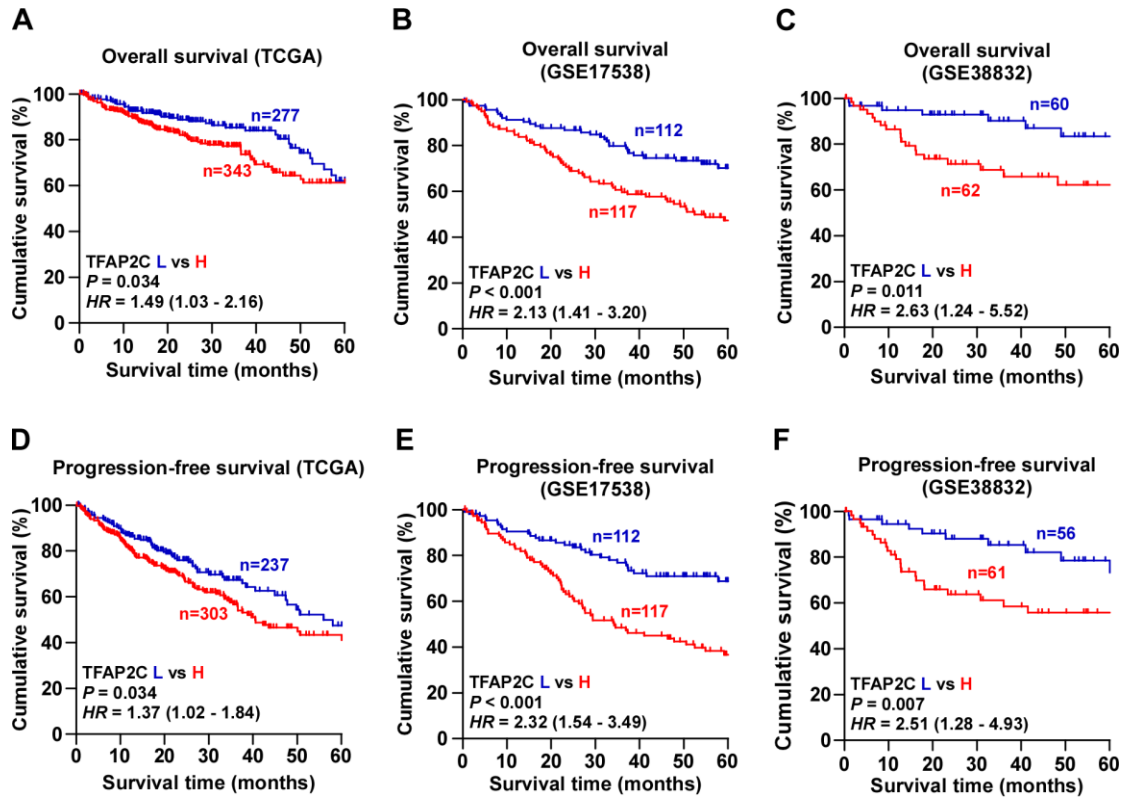

Supplement: Supplementary file 7 — Figure S2. Overexpression of TFAP2C is associated poor overall and progression-free survivals in CRC patients (A-C) Overall survival curves from the TCGA, GSE17538 and GSE38832 profiles for CRC patients stratified by high and low expression of TFAP2C. (D-F) Progression-free survival curves from the TCGA, GSE17538 and GSE38832 profiles for CRC patients stratified by high and low expression of TFAP2C. (PDF 233 kb) [file 13046_2018_683_MOESM7_ESM.pdf]

Supplemental Figure 3

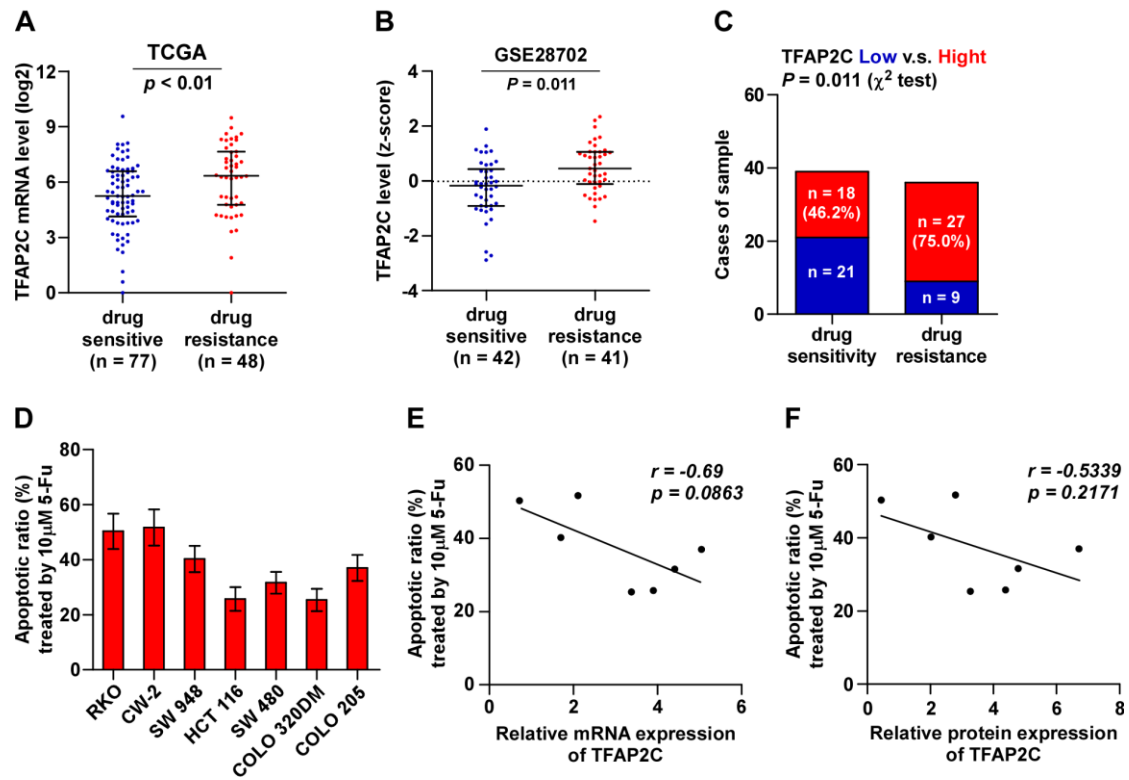

Supplement: Supplementary file 8 — Figure S3. Overexpression of TFAP2C is associated with poor chemotherapy response. (A and B) TFAP2C expression levels were much higher in CRC patients with poor chemotherapy response as assessed by analyzing the TCGA and GSE28702 CRC RNA sequencing datasets. (C) Percentages and number of samples showed high or low TFAP2C expression in CRC patients with different chemotherapy response in our CRC tissues. (D) Apoptotic ratio of CRC cells under treatment of 5-FU (20μm). (E and F) The correlation of TFAP2C mRNA (E) and protein (F) expression levels with apoptotic ratio in CRC cells after treated with 20μm 5-FU. (PDF 166 kb) [file 13046_2018_683_MOESM8_ESM.pdf]

Supplemental Figure 4

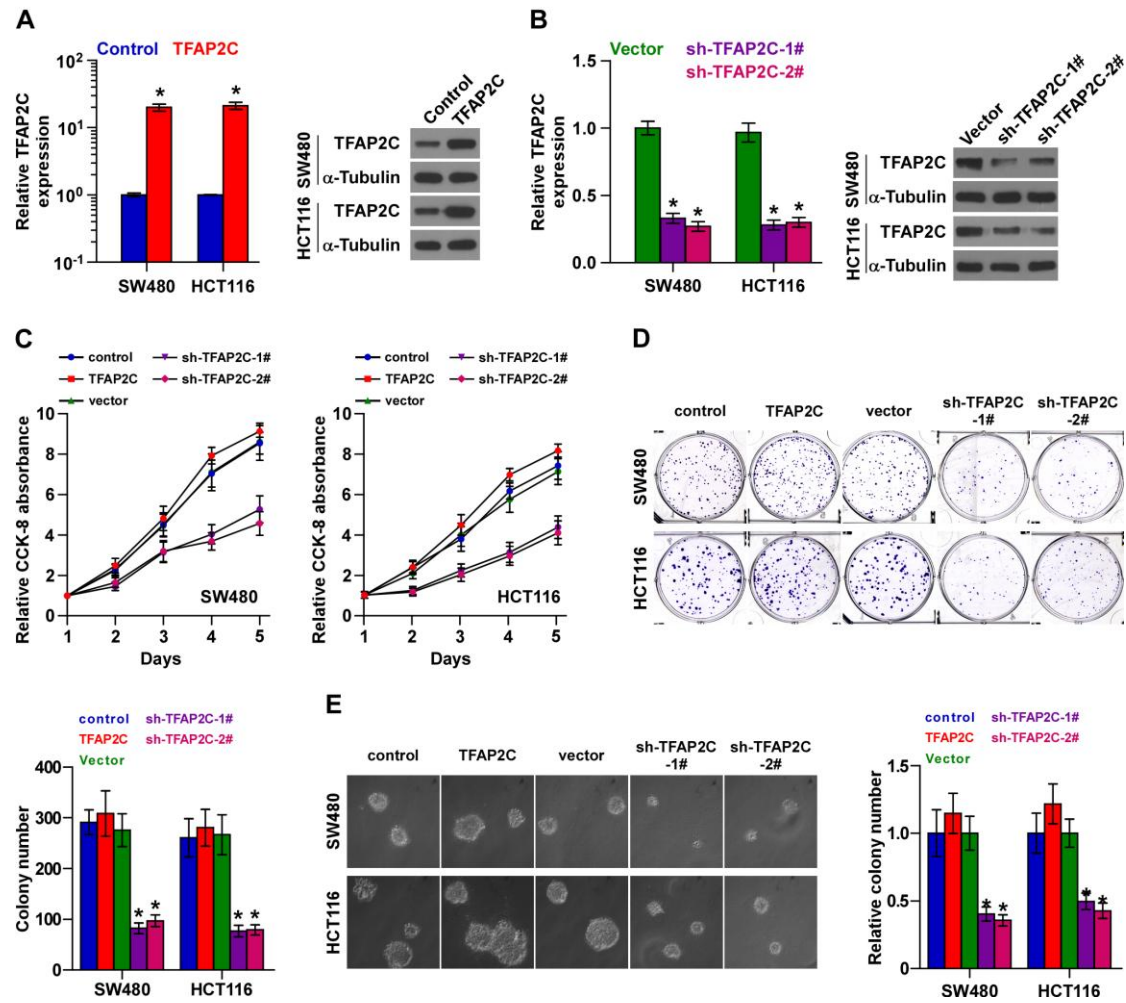

Supplement: Supplementary file 9 — Figure S4. Silencing TFAP2C inhibits proliferation ability of CRC cells. (A and B) Real-time PCR and Western blot of the indicated CRC cells transfected with TFAP2C -vector, TFAP2C, TFAP2C -RNAi-vector, TFAP2C -RNAi#1 and TFAP2C -RNAi#2. GAPDH was used as endogenous controls in RT-PCR and α-Tubulin was detected as a loading control in the Western blot. Each bar represents the mean values ± SD of three independent experiments. *P < 0.05. (C) CCK-8 assay revealed that silencing TFAP2C decreased the proliferation rate in CRC cells. Each bar represents the mean values ± SD of three independent experiments. *P < 0.05. (D) downregulation of endogenous TFAP2C reduced, the mean colony number in the colony formation assay. Each bar represents the mean values ± SD of three independent experiments. *P < 0.05. (E) Representative micrographs and colony numbers in the indicated group in the anchorage-independent growth assay. Each bar represents the mean values ± SD of three independent experiments. *P < 0.05. (PDF 167 kb) [file 13046_2018_683_MOESM9_ESM.pdf]

Supplemental Figure 5

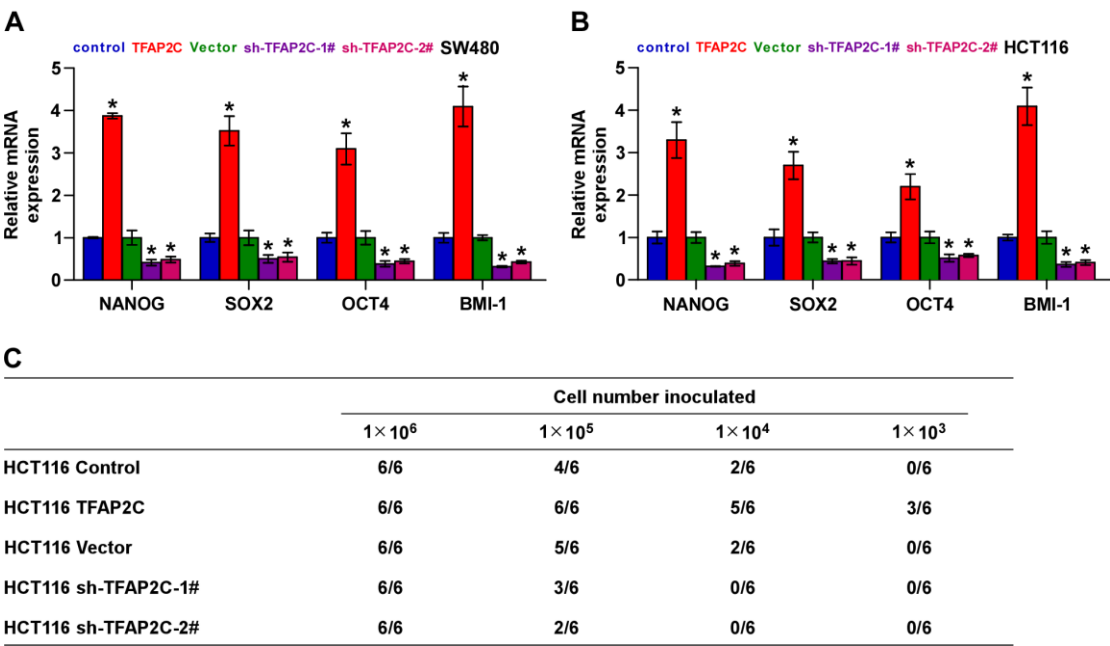

Supplement: Supplementary file 10 — Figure S5. (A and B) Real-time PCR analysis of OCT4A, SOX2, NANOG and BMI-1 expression in the indicated cells. GAPDH was used as the loading control. Error bars represent the mean ± S.D. of three independent experiments. *P < 0.05. (C) The formation number of tumor initiated by different amounts of HCT116 cells in nude mice. (PDF 106 kb) [file 13046_2018_683_MOESM10_ESM.pdf]

Supplemental Figure 6

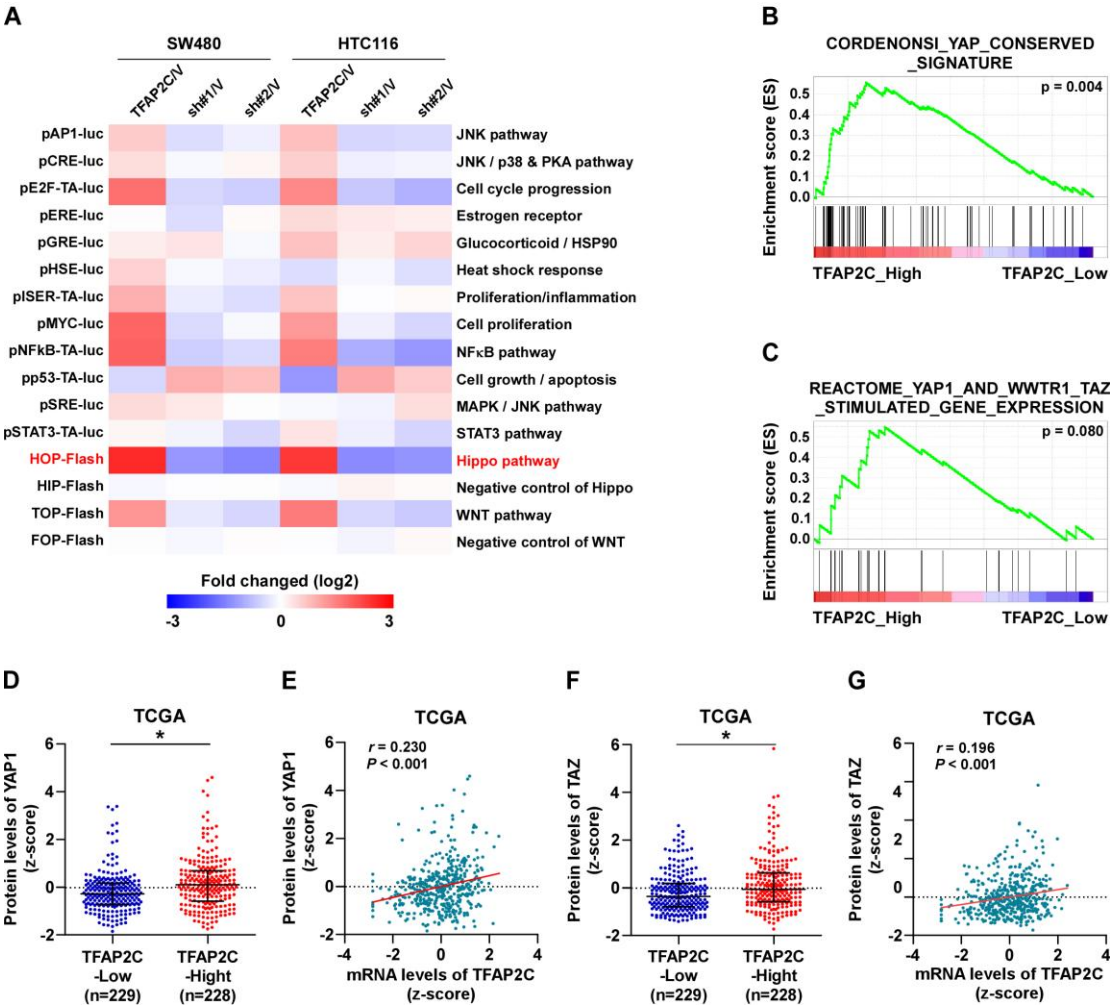

Supplement: Supplementary file 11 — Figure S6. (A) Activity of luciferase reporter constructs of several signaling pathway were examined in the TFAP2C-overexpressing or –silencing CRC cells. (B and C) TFAP2C expression level was positively associated with the YAP and TAZ-activated gene signatures. (D-G) TFAP2C expression level is positively associated with the protein expression levels of transcriptional co-activators YAP and TAZ of Hippo signaling pathway as assessed through CRC dataset from TCGA. (PDF 162 kb) [file 13046_2018_683_MOESM11_ESM.pdf]

## Supplemental Figure 7

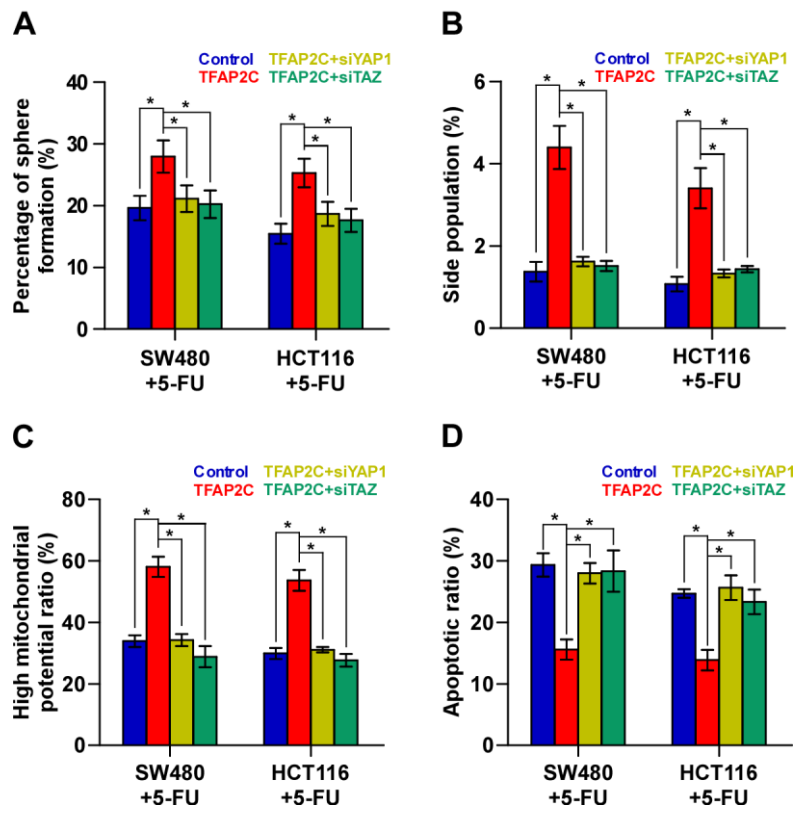

Supplement: Supplementary file 12 — Figure S7. (A and B) Individual silencing of YAP or TAZ attenuated the sphere formation ability and SP fraction in the TFAP2C-overexpressing CRC cells. *P < 0.05. (C and D) Individual silencing of YAP or TAZ reversed the effects of TFAP2C upregulation on mitochondrial potential and apoptotic ratio in CRC cells. *P < 0.05. (PDF 99 kb) [file 13046_2018_683_MOESM12_ESM.pdf]

Supplemental Figure 9

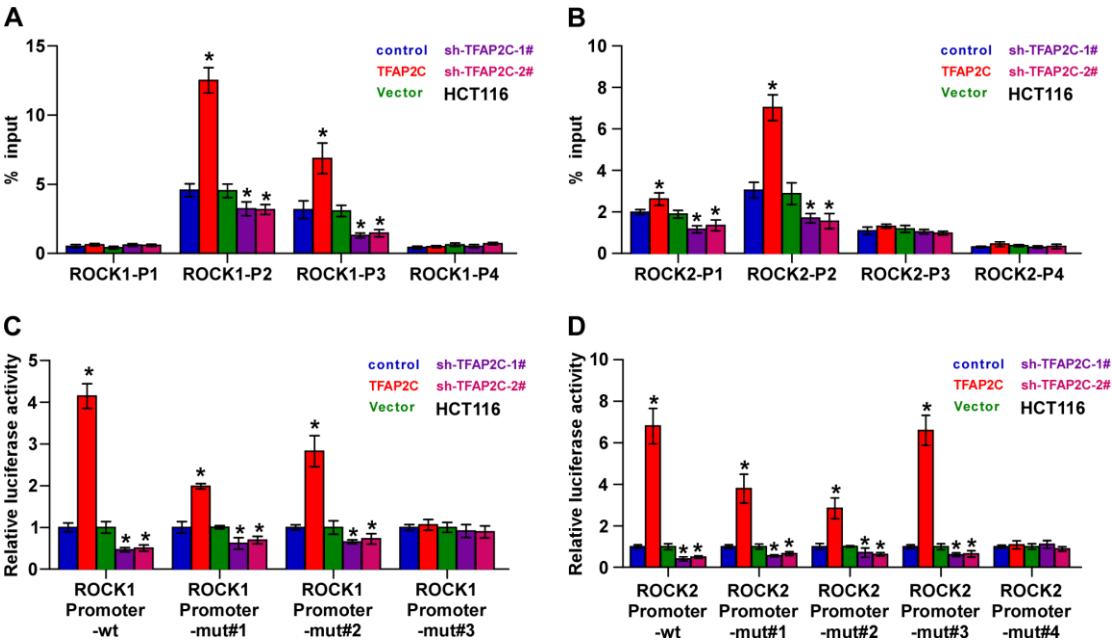

Supplement: Supplementary file 14 — Figure S9. (A and B) Analysis of ROCK1 and ROCK2 promoters physically associated with TFAP2C by using chromatin immunoprecipitation (ChIP) assay in the indicated HCT116 cells. *P < 0.05. (C and D) Relative luciferase activity of the indicated promoter vectors in the indicated HCT116 cells. *P < 0.05. (PDF 135 kb) [file 13046_2018_683_MOESM14_ESM.pdf]

Supplemental Figure 10

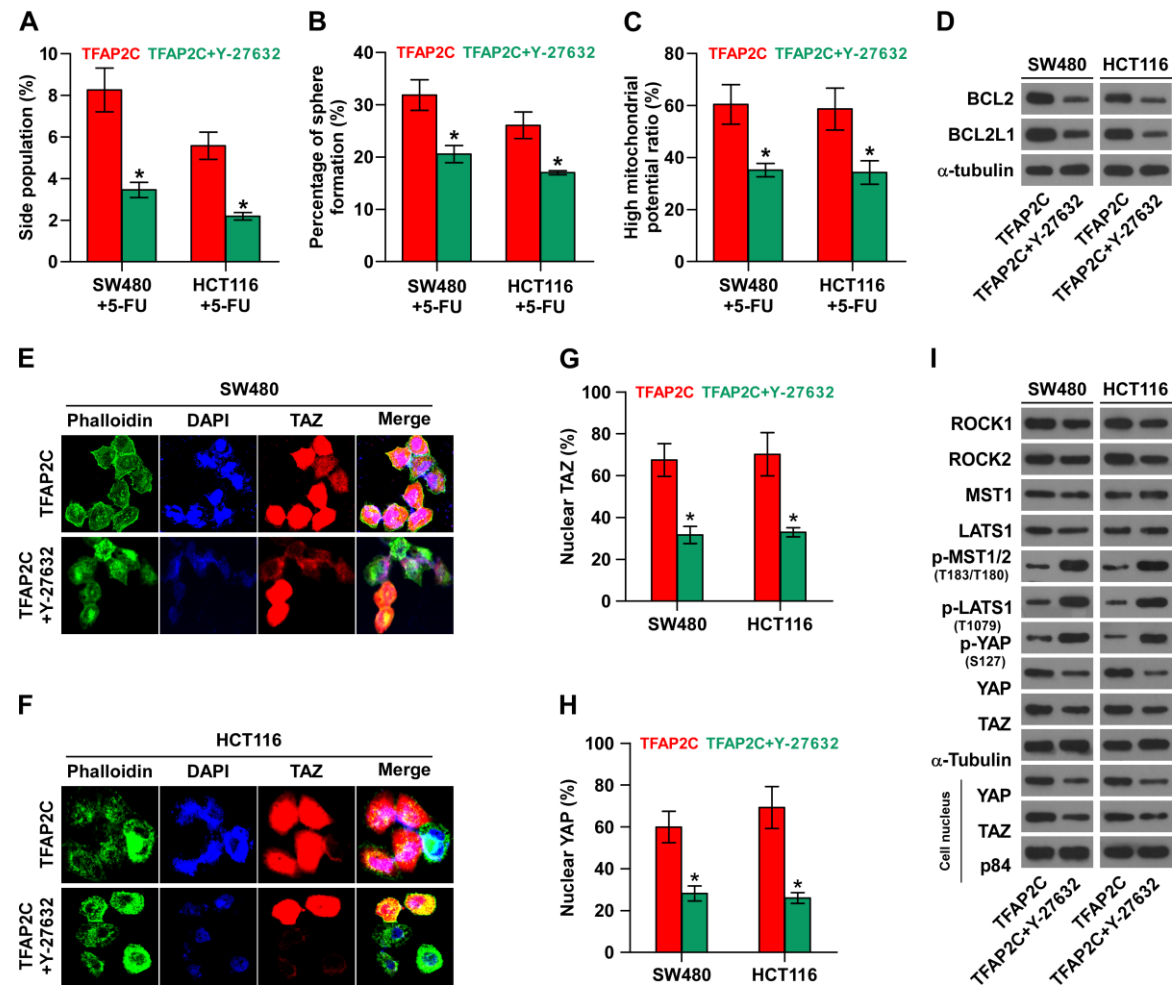

Supplement: Supplementary file 15 — Figure S10. (A-D) The specific inhibitor of ROCK1 and ROCK2, Y-27632, significantly repressed SP fraction, sphere formation ability, mitochondrial potential and BCL2, BCL2L1 expression in the TFAP2C-overexpressing CRC cells. (E and F) Representative immunofluorescent images of CRC cells were immunostained with YAP or TAZ antibody (red) or phalloidin (green) in the indicated CRC cells. (G and H) The percentage of nuclear TAZ+ (G) and nuclear YAP+ (H) cell number via immunostaining in the indicated groups. *P < 0.05. (I) Western blotting of ROCK1, ROCK2, p-MST1/2, MST1/2, p-LATS1, LAST1, p-YAP, YAP and TAZ expression, and nuclear YAP and TAZ expression in the indicated cells. α-tubulin and p84 were used as the loading control for cytoplasm and nucleus respectively. (PDF 383 kb) [file 13046_2018_683_MOESM15_ESM.pdf]

Supplemental Figure 11

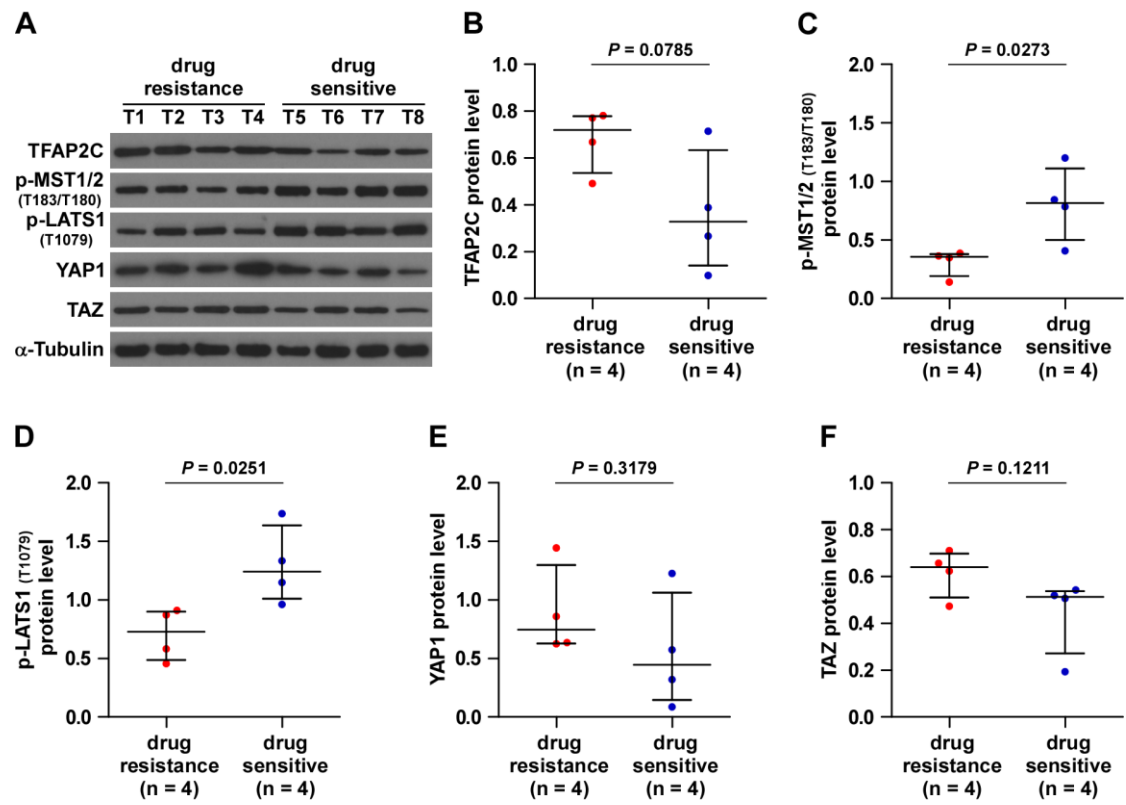

Supplement: Supplementary file 16 — Figure S11. Clinical relation of TFAP2C with Hippo signaling activity in human CRC tissues. (A) Analysis of TFAP2C expression with p-MST1/2, p-LATS1, YAP and TAZ expression in 4 resistant CRC tissues (T1-4) and 4 sensitive CRC tissues (T5-8). α-tubulin was used as loading control. (B-F) Relative expression levels of TFAP2C, p-MST1/2, p-LATS1, YAP and TAZ expression in CRC tissues.The expression levels of TFAP2C, p-MST1/2, p-LATS1, YAP and TAZ expression were quantified by densitometry using Image J, and normalized to the levels of α-tubulin respectively. The sample with the lowest expression of each protein was used as a standard. (PDF 191 kb) [file 13046_2018_683_MOESM16_ESM.pdf]
