# Supplementary material for: TFAP2C promotes stemness and chemotherapeutic resistance in colorectal cancer via inactivating hippo signaling pathway
Source: J Exp Clin Cancer Res. 2018 Feb 13;37:27. doi: 10.1186/s13046-018-0683-9 (PMC5812206; doi:10.1186/s13046-018-0683-9)

Supplemental Figure 8

A

There are 2 putative sites were predicted in ROCK1 promoter by JASPAR (chr6:18,690,100 - 18,692,700,Relative profile score threshold:90%)				
Modle ID	Strand	Score	Start	Predicted site sequence
MA0524.2	-	12.21	18,691,265	TGCCCGGAGGGA
MA0524.2	-	12.06	18,691,690	ACCCCCCAGGCA

B

There are 3 putative sites were predicted in ROCK2 promoter by JASPAR (chr6:11,482,400 - 11,486,050,Relative profile score threshold:90%)				
Modle ID	Strand	Score	Start	Predicted site sequence
MA0524.2	-	10.17	11,484,312	GGCCCCGAGGCT
MA0524.2	-	12.10	11,484,418	AGCCCCCAGGCG
MA0524.2	-	10.68	11,485,092	CGCCTCGGGGCC

C

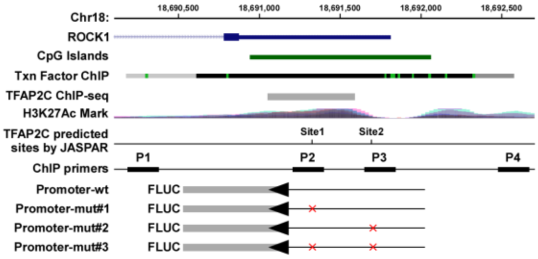

D

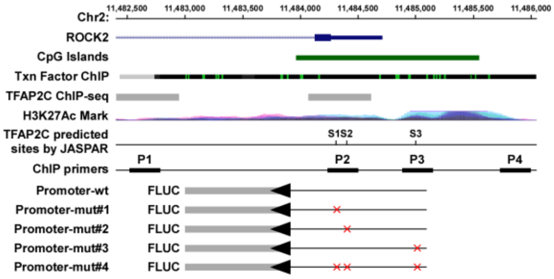

Supplement: Supplementary file 13 — Figure S8. (A-B) The putative binding sites of TFAP2C in ROCK1 and ROCK2 promoters by JASPAR. (C and D) Schematic representation of the promoter regions of ROCK1 and ROCK2 with the putative TFAP2C binding sites through UCSC. (PDF 171 kb) [file 13046_2018_683_MOESM13_ESM.pdf]
